# Engineered 3D vessel-on-chip using hiPSC-derived endothelial- and vascular smooth muscle cells

**DOI:** 10.1016/j.stemcr.2021.08.003

**Published:** 2021-09-02

**Authors:** Marc Vila Cuenca, Amy Cochrane, Francijna E. van den Hil, Antoine A.F. de Vries, Saskia A.J. Lesnik Oberstein, Christine L. Mummery, Valeria V. Orlova

**Affiliations:** 1 Department of Anatomy and Embryology, Leiden University Medical Center, Einthovenweg 20, 2333ZA Leiden, the Netherlands; 2 Department of Clinical Genetics, Leiden University Medical Center, 2333ZA Leiden, the Netherlands; 3 Department of Cardiology, Leiden University Medical Center, 2333ZA Leiden, the Netherlands

**Keywords:** hiPSC-derived endothelial cells, hiPSC-ECs, hiPSC-derived vascular smooth muscle cells, hiPSC-VSMCs, 3D vessel-on-chip, VoC, organ-on-chip, vessels-on-chip, microfluidics, functional readouts

## Abstract

Crosstalk between endothelial cells (ECs) and pericytes or vascular smooth muscle cells (VSMCs) is essential for the proper functioning of blood vessels. This balance is disrupted in several vascular diseases but there are few experimental models which recapitulate this vascular cell dialogue in humans. Here, we developed a robust multi-cell type 3D vessel-on-chip (VoC) model based entirely on human induced pluripotent stem cells (hiPSCs). Within a fibrin hydrogel microenvironment, the hiPSC-derived vascular cells self-organized to form stable microvascular networks reproducibly, in which the vessels were lumenized and functional, responding as expected to vasoactive stimulation. Vascular organization and intracellular Ca^2+^ release kinetics in VSMCs could be quantified using automated image analysis based on open-source software CellProfiler and ImageJ on widefield or confocal images, setting the stage for use of the platform to study vascular (patho)physiology and therapy.

## Highlights

• 3D VoC formed by hiPSC-ECs and hiPSC-VSMCs
• Vascular organization in 3D VoC formed by hiPSC-VSMC and primary mural cells
• Functional responses of hiPSC-VSMCs in 3D VoC
• Automated analysis of microvascular network morphology and Ca^2+^ release in VSMCs

## Graphical abstract

**Figure:**
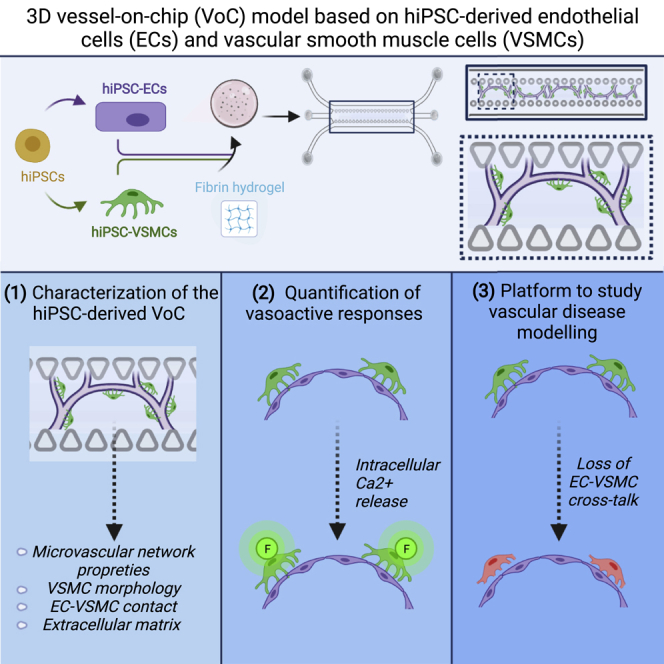

## Teaser

In this article, Orlova and colleagues describe the generation of an entirely human induced pluripotent stem cells (hiPSC)-derived vessel-on-chip composed with endothelial cells (ECs) and vascular smooth muscle cells (VSMCs). Changes in microvascular network architecture and intracellular Ca^2+^ release kinetics could be quantified during vascular organization or upon drug treatment providing a useful platform to study vascular development and disease.

## Introduction

Crosstalk between endothelial cells (ECs) and mural cells (pericytes and vascular smooth muscle cells [VSMCs]) is pivotal for proper function of many blood vessels. Aberrant EC-mural cell crosstalk often leads to vascular diseases that range from hypertension, atherosclerosis, vascular calcification, and coronary artery disease to stroke and other conditions (Owens et al., 2004). Animal models, including genetically modified mice, are widely used to study vascular development and disease, but these do not always capture patient phenotypes unless the mutations are homozygous deletions, and differences associated with the genetic background and susceptibility are not evident in mice (Berry et al., 2019; Van Norman, 2020). Human induced pluripotent stem cells (hiPSCs) generated from heathy individuals and patients are a useful source of vascular cells and they do reflect the genetic background of the individual from whom they are derived (Samuel et al., 2015). Several methods have been described to generate ECs and VSMCs from hiPSCs and some have already been used to model vascular disease-specific abnormalities (Cochrane et al., 2019).

Nevertheless, and despite recent advances, many current *in vitro* models of blood vessels fail to emulate the integrated, complex and multicell-type composition of the human vasculature and do not include a mimic of blood flow (Duval et al., 2017). To address this, microfluidic devices have been engineered that do incorporate these features and provide the environment for the formation of multi-cell type 3D tissues and vessels-on-chip (VoC) (Tronolone and Jain, 2021). Typically, cells incorporated in these microphysiological devices are derived from non-human sources, human (tumor) cell lines, or directly from primary human tissue. Primary human cells provide the closest mimic to human blood vessels but are of limited availability and of variable genetic origin (Tronolone and Jain, 2021). While hiPSC derivatives are now regarded as an alternative, they have so far largely been used in combination with primary cells in microfluidic chips. For example, human primary ECs, or hiPSC-ECs have been combined with human primary mural cells (Campisi et al., 2018; van Dijk et al., 2020; van Duinen et al., 2019) but not with mural cells derived from hiPSC, precluding opportunities to replicate (patient-specific) vascular diseases originating in the mural cells.

Here, we overcome these limitations by incorporating hiPSC-ECs with hiPSC-VSMCs in entirely hiPSC-based VoCs. We have previously described robust protocols to derive ECs and VSMCs from multiple healthy hiPSC lines with little batch-to-batch variability (Halaidych et al., 2018, 2019; Orlova et al., 2014a, 2014b). The functionality of hiPSC-VoC was compared with similar VoCs containing hiPSC-ECs and human primary mural cells of the same development origin, namely human brain vascular smooth muscle cells (HBVSMCs) and primary human brain vascular pericytes (HBVPs). In all cases, we showed the microenvironment in the microfluidic device supported the formation of a 3D perfusable, self-assembled microvascular network. We optimised the culture conditions and developed an automated quantification framework of the vascular network, mural cell morphology, EC-mural cell interaction and extracellular matrix (ECM) composition. Finally, we demonstrated a functional application: the automated quantification of vasoactive responses.

## Results

### Characterization of the VoC model

**Figure 1:**
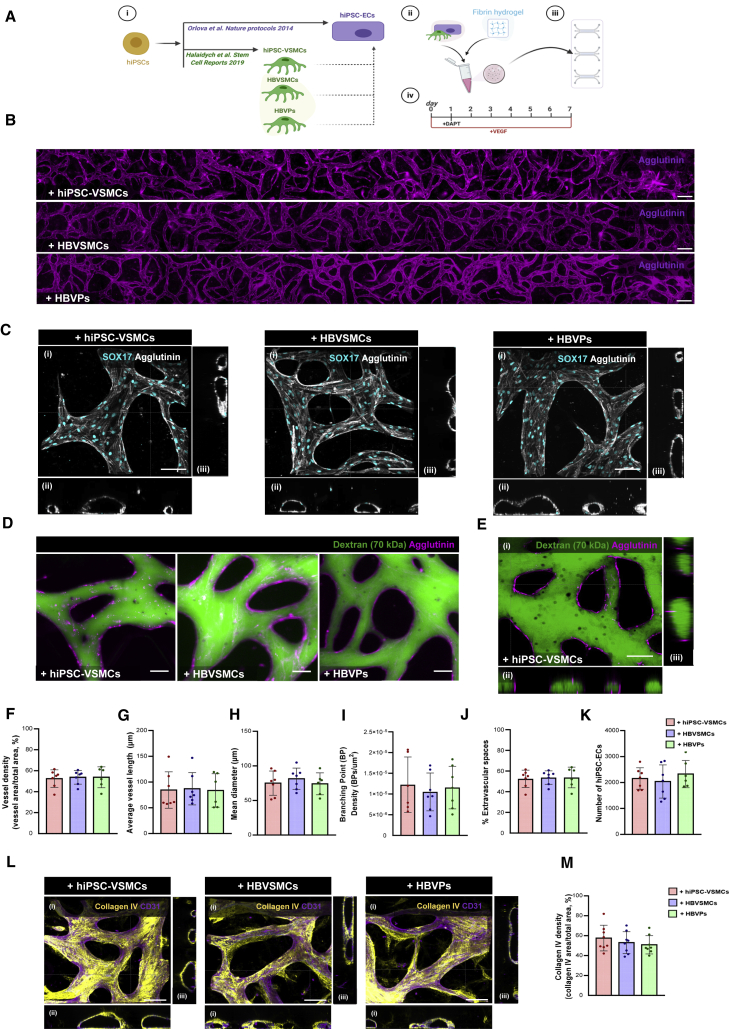
Characterization of VoC (A) Schematic of the VoC protocol. hiPSC-ECs were cultured with hiPSC-VSMCs, HBVSMCs, or HBVPs (i). Cells were mixed in a fibrin hydrogel (ii) and injected into (AIM Biotech) microfluidic chips (iii). EGM-2 was supplemented with VEGF (50 ng/mL), chips were refreshed daily for 7 days. EGM-2 was also supplemented with DAPT (10 μM) on day 1 for 24 h (iv). (B) Representative immunofluorescence images of microvascular network showing hiPSC-EC (magenta; agglutinin) vessels spanning the complete length of the microfluidic channel. Images showing hiPSC-ECs cultured with hiPSC-VSMCs, HBVSMCs, or HBVPs, respectively (10×). Scale bars, 200 μm. (C) Representative confocal images of microvascular network showing hiPSC-ECs (gray; agglutinin) and hiPSC-EC nuclei (cyan; SOX17). Images displaying xyz (i), xy (ii), and yz cross-sectional perspectives (iii). Images showing hiPSC-ECs cultured with hiPSC-VSMCs, HBVSMCs, or HBVPs, respectively (40×). Scale bars, 100 μm. (D) Representative Immunofluorescence images showing hiPSC-ECs (magenta; agglutinin) and perfusion of 70 kDa FITC-Dextran (green). Images showing hiPSC-ECs cultured with hiPSC-VSMCs, HBVSMCs, or HBVPs, respectively (10×). Scale bars, 50 μm. (E) Representative confocal images showing hiPSC-ECs (magenta; agglutinin) and perfusion of 70 kDa FITC-Dextran (green) in hiPSC-VoC. Images displaying xyz (i), xy (ii), and yz cross-sectional perspectives (iii) (40×). Scale bars, 100 μm. (F–K) Quantification of vessel density (%) (F), average vessel length (μm) (G), mean diameter (μm) (H), branching point (BP) density (BPs/μm^2^) (I) extravascular spaces (%) (J), and number of hiPSC-ECs (K), from hiPSC-ECs cultured with hiPSC-VSMCs, HBVSMCs, or HBVPs, respectively, are shown. Data are shown as ±SD from N = 3, n = 6; three independent experiments with two microfluidic channels per experiment. (L) Representative confocal images of microvascular network showing hiPSC-ECs (magenta; CD31) and ECM (yellow; collagen IV). Images displaying xyz (i), xy (ii), and yz cross-sectional perspectives (iii). Images showing hiPSC-ECs cultured with hiPSC-VSMCs, HBVSMCs, or HBVPs, respectively (40×). Scale bars, 100 μm. (M) Quantification of collagen IV density (%) from hiPSC-ECs cultured with hiPSC-VSMCs, HBVSMCs, or HBVPs, respectively, are shown. Data are shown as ±SD from N = 3, n = 8; three independent biological replicates with two to three microfluidic channels per experiment. One-way ANOVA with Tukey's multiple comparison. See also Figures S1 and S2A–S2D and Video S1.

Using the commercially available AIM Biotech 3D cell culture chips, hiPSC-ECs (Halaidych et al., 2018; Orlova et al., 2014a, 2014b) were combined with hiPSC-VSMCs (Halaidych et al., 2019), HBVSMCs or HBVPs (Figure 1Ai) in a fibrin hydrogel (Figure 1Aii) and the cell/gel mix was injected into the middle channel of the microfluidic chip (Figure 1Aiii). As a control, hiPSC-ECs in a fibrin hydrogel without mural cells were used (Figure S1). Endothelial growth medium-2 (EGM-2), supplemented with vascular endothelial growth factor (VEGF) (50 ng/mL), was used to support microvascular network formation. Microfluidic chips were perfused through gravity-driven flow by adding 100 μL of medium to the inlet and 50 μL to the outlet of each medium channel. The gravity-driven flow was re-established every 24 h allowing medium exchange in the microfluidic chip (Table S1). On day 1, after hiPSC-ECs had begun to self-organize, γ-secretase inhibitor DAPT (10 μM) was added to the medium for 24 h to promote hiPSC-EC sprouting (Figure 1Aiv). Vacuoles started to appear in hiPSC-ECs 12–24 h after seeding (Figure S2A, white arrowheads) followed by proliferation and remodeling up to 72 h. An interconnected microvascular network formed as early as day 2 (Figure S2A) and this spanned the complete microfluidic channel by day 7 (Figures 1B, S1A, and S2A). All combinations of mural cells with hiPSC-ECs resulted in vascular lumen formation (Figure 1C). Furthermore, these lumenized networks were perfusable by fluorescent beads (10 μm) or FITC-Dextran (70 kDa) (Figures 1D and 1E; Video S1) under gravity-driven flow. Microvascular networks formed in the presence of hiPSC-VSMCs or primary mural cells showed similar morphologies, with no significant difference in vessel density (%, Figure 1F), average vessel length (μm, Figure 1G), mean vessel diameter (μm, Figure 1H), branching point (BP) density (BP/μm^2^, Figure 1I), extravascular spaces (%, Figure 1J), or number of hiPSC-ECs (Figure 1K). In contrast, microvascular networks formed by hiPSC-ECs alone were less organized than microvascular networks with hiPSC-VSMCs (Figure S1A). Although lumen formation was observed in microvascular networks formed using only hiPSC-ECs, these appeared irregular and broken (Figure S1B). The instability of microvascular networks without hiPSC-VSMCs was also evidenced by increased leakage of FITC-Dextran from the vessel network (70 kDa; Figure S1I). There were, however, no significant differences in vessel density (%, Figure S1C), branching point (BP) density (BP/μm^2^, Figure S1F), extravascular space (%, Figure S1G), or number of hiPSC-ECs (Figure S1H) in microvascular networks formed either with or without hiPSC-VSMCs. By contrast, average vessel length (μm, Figure S1D) and mean vessel diameter (μm, Figure S1E) were significantly reduced or increased respectively in microvascular networks formed from hiPSC-ECs alone compared with microvascular networks formed in the presence of hiPSC-VSMCs. No significant differences were found in ECM deposition, evidenced by changes in the relative density of collagen IV, between any cell combinations (Figures 1L, 1M, S1J, and S1K). Furthermore, the presence of fibronectin was confirmed by immunostaining in microvascular networks formed with mural cells (Figure S2D). Finally, long-term culture of hiPSC-ECs in VoC with mural cells demonstrated that the microvascular network architecture was stable and underwent continuous increase in vessel density over 21 days (Figures S2A–S2C), although we observed an increase in density of vessel networks formed with primary HBVSMCs on day 21 (Figures S2A–S2C).

Video S1. Perfusion of fluorescent beads (top panels) and FITC-Dextran (70 kDa) (bottom panels), related to Figure 1

### Characterization of hiPSC-VSMCs and primary mural cells in the VoC model

**Figure 2:**
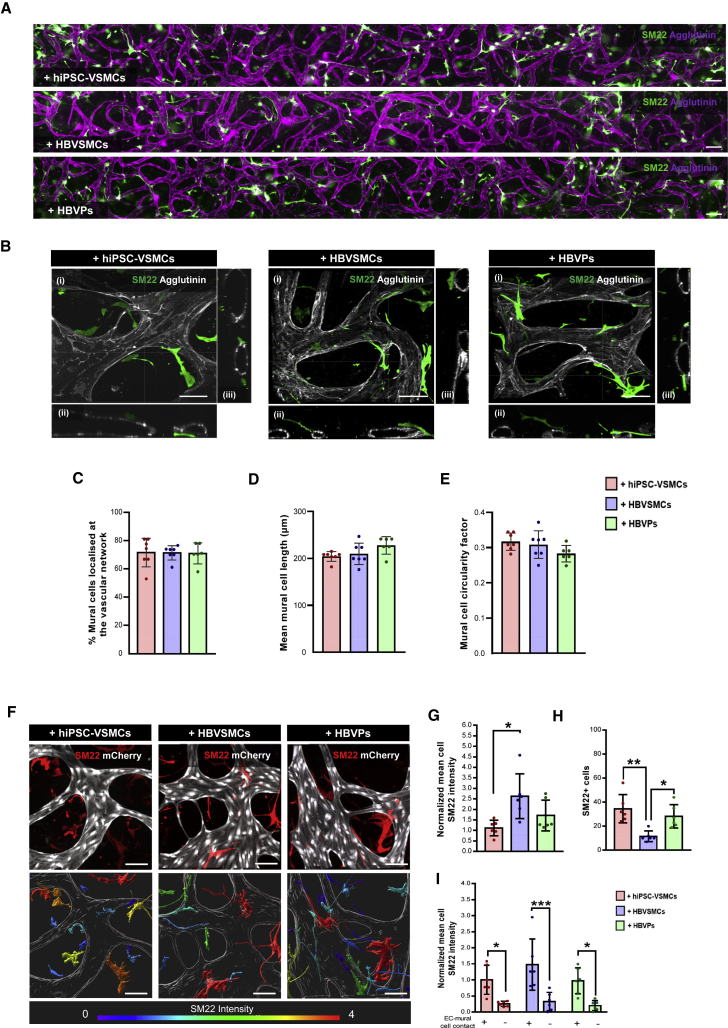
Quantitative assessment of the structural proprieties of hiPSC-VSMCs and primary mural cells in VoC (A) Representative immunofluorescence images of microvascular network showing the hiPSC-ECs (magenta; agglutinin) and mural cells (green; SM22) derived vasculature unit spanning the complete length of microfluidic channel. Images showing hiPSC-ECs cultured with hiPSC-VSMCs, HBVSMCs, or HBVPs, respectively (10×). Scale bars, 200 μm. (B) Representative confocal images of microvascular network showing hiPSC-ECs (gray; agglutinin) and mural cells (green; SM22). Images displaying xyz (i), xy (ii), and yz cross-sectional perspectives (iii). Images showing hiPSC-ECs cultured with hiPSC-VSMCs, HBVSMCs, or HBVPs, respectively (40×). Scale bars, 100 μm. (C–E) Quantification of the percentage of mural cells associated with the hiPSC-EC lumen (% mural cells localized at the vascular network) (C), mean mural cell length (μm) (D), and mural cell circularity factor (the circle is 1) (E) in hiPSC-VSMCs, HBVPs, and HBVSMCs. Data are shown as ±SD from N = 3, n = 6; three independent experiments with two microfluidic channels per experiment. (F) (Top) Representative confocal images of microvascular network showing hiPSC-ECs (gray; mCherry) and mural cells (red; SM22). (Bottom) Representative surface-rendered objects of confocal images showing microvascular network (gray; mCherry) and mural cells (colour-coded scale representing SM22 intensity). Images showing hiPSC-ECs cultured with hiPSC-VSMCs, HBVSMCs, or HBVPs, respectively (40×). Scale bars, 100 μm. (G–I) Quantification of normalized mean cell SM22 intensity (G), number of SM22 + cells (H), and normalized mean cell SM22 intensity of mural cells in contact with hiPSC-ECs (I) in hiPSC-VSMCs, HBVPs and HBVSMCs. Intensity was normalized to hiPSC-VSMCs. Data are shown as ±SD from N = 3, n = 6; three independent experiments with two microfluidic channels per experiment. One-way ANOVA (C–E and G–H) and two-way ANOVA (I) with Tukey's multiple comparison. ^∗^p < 0.05, ^∗∗^p < 0.001, ^∗∗∗^p < 0.0001. See also Figures S2E and S2F.

hiPSC-VSMCs, HBVSMCs, and HBVPs self-organized and self-oriented toward the developing hiPSC-EC microvascular networks, as early as day 1 (Figure S2E). On day 7, all mural cells were located at extravascular positions along the entire length of the microvascular network (Figure 2A), surrounding hiPSC-EC lumens (Figure 2B). No significant difference was observed in the percentage of hiPSC-VSMCs, HBVSMCs, or HBVPs associated with the hiPSC-EC lumen (% mural cells localized at the vascular network, Figure 2C). Quantification of mural cell morphology also showed no significant difference in the cell length (μm, Figure 2D) or cell circularity (Figure 2E). Analysis of the contractile marker SM22 in mural cells in microvascular networks showed a significantly lower normalized mean cell intensity in hiPSC-VSMCs compared with HBVSMCs (Figures 2F and 2G) while the total number of SM22 + cells was significantly lower in microvascular networks formed with HBVSMCs (Figure 2H). Notably, we also observed that all mural cells displayed significantly higher SM22 staining intensity when in contact with hiPSC-ECs (Figures 2F and 2I), which indicated that heterotypic cell-cell contact in VoC culture could further promote mural cell maturation. Furthermore, long-term hiPSC-VoC culture resulted in an increase over time in the percentage of mural cells located close to the hiPSC-EC vessel wall (Figures S2E and S2F).

### Assessment of hiPSC-VSMC Ca^2+^ dynamics in the VoC model

**Figure 3:**
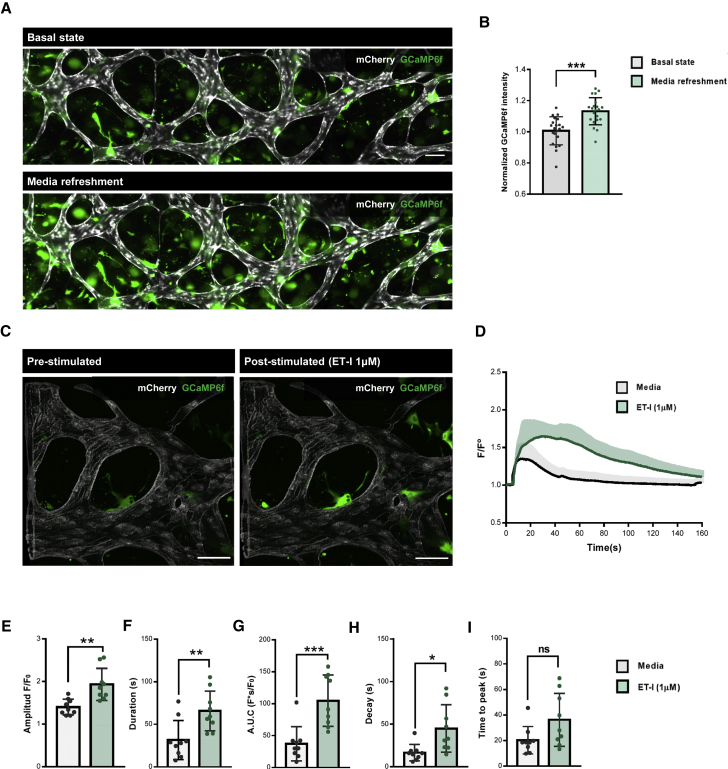
Analysis of hiPSC-VSMCs Ca^2+^ dynamics in VoC (A) Representative immunofluorescent images of intracellular Ca^2+^ fluorescence showing hiPSC-ECs (gray; mCherry) and hiPSC-VSMCs (green; GCaMP6f) without- (basal state) and after EGM-2 refreshment on day 7 (10×). Scale bar, 100 μm. (B) Normalized GCaMP6f intensity at day 7. GCaMP6f intensity was normalized to the condition prior to EGM-2 refreshment (basal state). Data are shown as ±SD of N = 3, n = 21; three independent experiments with seven microfluidic channels per experiment. (C) Representative confocal images of intracellular Ca^2+^ fluorescence showing with hiPSC-ECs (gray; mCherry) and hiPSC-VSMCs (green; GCaMP6f) in pre- and post-stimulated (ET-I, 1 μM) states (20×). Scale bar, 100 μm. (D) Normalized average fluorescence intensity F/F_0_ in hiPSC-VSMCs expressing GCaMP6f. Medium channels were gravity-flow perfused with EGM-2 alone or containing ET-I (1 μM). Stimulation time point is set as t = 5(s). Data are shown as ±SD of N = 3, n = 9; three independent experiments with three microfluidic channels per experiment. (E–I) Ca^2+^ transient parameters: amplitude F/F_0_ (E), duration (s) (F), area under the curve (AUC, F^∗^s/F_0_) (G), time to peak (s) (H), and decay (s) (I) of channels gravity-flow perfused with EGM-2 alone or containing ET-I (1 μM). Data are shown as ±SD of N = 3, n = 9; three independent experiments with three microfluidic channels per experiment. Paired (B) Student's t test. Wilcoxon-Mann-Whitney test (E–I). ^∗^p < 0.05, ^∗∗^p < 0.001, ^∗∗∗^p < 0.0001; ns, not significant. See also Figure S3 and Video S2.

To further assess the functionality of hiPSC-VSMCs in the hiPSC-VoC, we measured intracellular Ca^2+^ release in hiPSC-VSMCs engineered to express an ultra-sensitive Ca^2+^ sensor (GCaMP6f) (Chen et al., 2013). First, hiPSC-derived neural crest (NC) intermediates were transduced with a lentiviral vector (LV) expressing GCaMP6f (Figure S3A). Transduction of hiPSC-NC cells with LVs encoding either enhanced green fluorescent protein or GCaMP6f did not change expression of the surface marker CD271 (Figure S3B). hiPSC-VSMCs engineered to express GCaMP6f showed no intracellular Ca^2+^ release upon perfusion with medium only (pre-stimulated, Figures S3C and S3D) and similar intracellular Ca^2+^ release upon stimulation with the vasoconstrictor endothelin-I (ET-I) (post-stimulated, Figures S3C and S3D) to Fluo-4-labeled hiPSC-VSMCs (Halaidych et al., 2019). Next, intracellular Ca^2+^ release in hiPSC-VSMCs in the microvascular network was examined prior to- (basal state) and after medium refreshment on day 7. Significantly higher GCaMP6f fluorescence was observed across the entire microvascular network after medium refreshment (Figures 3A and 3B; Video S2). We then combined medium refreshment with ET-1 stimulation (1 μM, Figure 3C; Video S2). To compare the divergence in Ca^2+^ responses, the average fluorescence intensity of regions of interest over time (F/F_0_) was examined by adapting previous methods (Halaidych et al., 2019) (Figure 3D). Comparison of Ca^2+^ kinetic parameters, measured at the half-maximum level (F/F0)_max_, showed significantly higher amplitudes (F/F_0_, Figure 3E), intensity over time (AUC [F^∗^s/F0], Figure 3F) and duration, and slower decay of the Ca^2+^ transient without changes in the time to peak (s, Figures 3G–3I) upon ET-I stimulation.

Video S2. Intracellular Ca2+ release in hiPSC-VSMC in VoC upon medium refreshment (left panel) and upon ET-I stimulation (right panel), related to Figure 3

### Modeling hiPSC-derived EC-VSMC crosstalk in the VoC model

**Figure 4:**
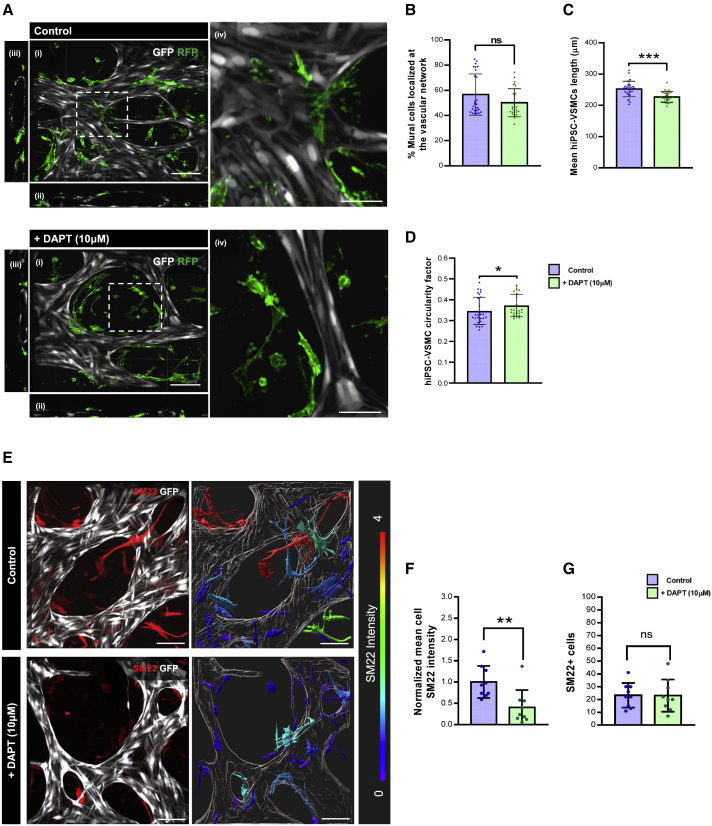
Modeling loss of EC-VSMC crosstalk in VoC (A) Representative confocal images of microvascular network showing in hiPSC-ECs (gray; GFP) and hiPSC-VSMCs (green; RFP) in control and DAPT (10 μM) supplemented conditions. Images displaying xyz (i), xy, (ii), yz cross-sectional perspectives (iii), and enlargements of white framed areas (iv) (40×). Scale bars, 100 μm in (i–iii) and 50 μm in (iv). (B–D) Quantification of the percentage of hiPSC-VSMCs associated with the hiPSC-EC lumen (% mural cells localized at the vascular network) (B), mean hiPSC-VSMCs length (μm) (C), and hiPSC-VSMC circularity factor (the circle is 1) (D) in control and DAPT (10 μM) supplemented conditions at day 7. Data are shown as ±SD from N = 3, n = 27; three independent experiments with nine microfluidic channels per experiment. (E) (Left) Representative confocal images of microvascular network showing hiPSC-ECs (gray; mCherry) and hiPSC-VSMCs cells (red; SM22). (Right) Representative surface-rendered objects of confocal images showing microvascular network (gray; mCherry) and hiPSC-VSMCs (colour-coded scale representing SM22 intensity) in control and DAPT (10 μM) supplemented conditions (40×). Scale bars, 100 μm. (F and G) Quantification of normalized mean cell SM22 intensity (F) and number of SM22 + cells (G). Intensity was normalized to control condition. Data are shown as ±SD from N = 3, n = 9; three independent experiments with three microfluidic channels per experiment. Wilcoxon-Mann-Whitney test. ^∗^p < 0.05, ^∗∗^p < 0.001, ^∗∗∗^p < 0.0001; ns, not significant. See also Figure S4.

To demonstrate the potential utility of hiPSC-VoC for disease modeling, we examined the loss of EC-VSMC crosstalk upon blocking NOTCH signaling using the small-molecule γ-secretase inhibitor DAPT (10 μM). The addition of DAPT (10 μM, day 5–7) affected overall microvascular network architecture (Figure S4A) with significant changes in the mean vessel diameter although vessel density was similar between the groups (Figures S4B and S4C). DAPT supplementation had no significant effect on the percentage of hiPSC-VSMCs associated with the hiPSC-EC lumen (% mural cells localized at the vascular network, Figures 4A and 4B). However, hiPSC-VSMCs showed a significant decrease in the cell length (μm, Figure 4C) with a significant increase in cell circularity (Figure 4D). Analysis of the contractile marker SM22 in hiPSC-VSMCs showed a significantly lower normalized mean SM22 intensity (Figures 4E and 4F) while no change in the total number of SM22 + cells was observed, following addition of DAPT to the cultures (Figure 4G).

## Discussion

This report describes the generation of hiPSC-derived microvascular networks composed of hiPSC-ECs and hiPSC-VSMCs. We showed that hiPSC-ECs form an interconnected microvascular network with perfusable lumens and ECM deposits as in previous microfluidic studies based on other cell sources (Belair et al., 2015; Campisi et al., 2018). We demonstrate that although hiPSC-ECs alone can form a microvascular network, inclusion of mural cells facilitates hiPSC-EC self-organization and supports vessel stability. Much like primary mural cells, hiPSC-VSMCs assumed positions surrounding the vascular wall, supporting the vessel and maintaining its functionality in 3D. Although we did not find any morphological differences in microvascular network organization and mural cell morphology between hiPSC-VSMCs and primary mural cells, expression of the contractile marker SM22 did differ between hiPSC-VSMCs and HBVSMCs. hiPSC-VSMCs and HBVPs showed lower SM22 staining intensities and higher total cell numbers in VoC culture. This could indicate that hiPSC-VSMCs are less differentiated in VoC culture. Notably, by incorporating and monitoring fluorescently tagged hiPSC-derived vascular cells (Roberts et al., 2017), we showed that the most important steps of vascular network formation and remodeling occurred in the first 7 days of VoC culture. We also showed that these microvascular networks are stable for up to 3 weeks. Vessel density increased over time, although not much remodeling occurring beyond day 7 of culture. Specifically, microvascular networks formed with primary HBVSMCs showed the highest increase in vessel density, indicating that an increase in hiPSC-VSMC number observed on day 7 might be advantageous for supporting long-term VoC culture. Moreover, we demonstrated that hiPSC-VSMCs in the VoC were responsive to vasoactive stimulation by quantifying changes in Ca^2+^ kinetic parameters. We noted that hiPSC-VSMCs were activated simply after medium refreshment, an important consideration for proper experimental control. It seemed likely this was mediated by factors in the fresh media since shear-stress during gravity-mediated flow in the VoC was too slow to be stimulatory. In addition, we confirmed more pronounced and coordinated hiPSC-VSMC intracellular Ca^2+^ release following addition of the vasoconstrictor ET-I.

Conventional preclinical models have shown low success in accurately predicting drug efficacy and toxicity in human trials (Van Norman, 2020). We considered it essential therefore to provide some evidence that hiPSC-VoC responded to drugs as expected and that this property was conserved across different healthy hiPSC lines and batches. This indeed was the case: under conditions of vessel maturation where the NOTCH signaling pathway coordinates heterologous cell-cell crosstalk and maintains vessel integrity, we showed that these features were disrupted in the hiPSC-VoC after inhibition by DAPT on days 5–7. In particular, DAPT addition appeared to reduce acquisition of a contractile-like identity by hiPSC-VSMCs. This notion was supported by reduced expression the contractile marker SM22 and a less elongated morphology after EC-VSMC crosstalk inhibition by DAPT. We also observed gradual reversal of vascular stability and signs of vessel regression, reflecting what is known about dysfunctional EC-VSMC crosstalk in developed vessels *in vivo* (Kerr et al., 2016).

In summary, we have demonstrated several advantages of this hiPSC-VoC model above existing models: (1) the possibility of studying genetic vascular diseases with patient-specific lines; (2) evaluation of real-time changes in vascular structure and function by incorporating fluorescently tagged cells; (3) the ability to measure and quantify effects of drugs affecting EC-mural cell crosstalk with view to modulating vessel stability and integrity. Nevertheless, the model could be further improved by: (1) introducing physiological (rather than gravity-driven) flow which would recapitulate vessel shear forces and geometry in healthy and disease environments and (2) the addition of other hiPSC-derived cells such as monocytes or astrocytes which would allow mimicking of (isogenic) inflammatory reactions or features of the brain.

In conclusion, the hiPSC-VoC model described in this paper will be useful to study and quantify changes in the vascular architecture and function during vascular development or upon drug treatment. Clinically, this may translate into a better understanding of vascular disease conditions and predicting drug efficiency.

## Experimental procedures

Full details are provided in supplemental experimental procedures.

### hiPSC lines

Research on hiPSC was approved by the medical ethical committee at Leiden University Medical Center, the Netherlands. A detailed list of the hiPSC lines and batches used for each experiment is provided in Table S2.

### Differentiation of hiPSC-ECs and hiPSC-VSMCs

hiPSC differentiation to ECs was performed as described previously (Orlova et al., 2014a, 2014b). hiPSC differentiation to VSMC was performed as previously described (Halaidych et al., 2019).

### Setting up VoCs

hiPSC-ECs and mural cells were prepared prior to incorporation in VoCs as described in supplemental experimental procedures. Commercially available microfluidic chips with one gel channel and two media channels (AIM Biotech) were used. Cells were resuspended and combined to obtain 10 × 10^6^ hiPSC-ECs/mL and 2 × 10^6^ mural cells/mL (5:1 ratio). Three different mural cell suspensions were tested in combination with hiPSC-ECs: (1) hiPSC-VSMCs, (2) HBVSMCs, and (3) HBVPs. Cell were resuspended in EGM-2 supplemented with Thrombin (4 U/mL) and then gently mixed with fibrinogen (final concentration 3 mg/mL, Sigma) at 1:1 vol ratio. Cell/hydrogel mixture was quickly loaded into the middle gel-loading channel of the microfluidic chip. Chips were incubated at room temperature for 15 min before the addition of EGM-2 supplemented with VEGF (50 ng/mL) to both flanking media channels. The γ-secretase inhibitor DAPT (10 μM) was also added to the medium on day 1 for 24 h. Gravity-driven flow was induced by the addition of 100 μL medium to the right media ports and 50 μL media to left media ports. Medium was refreshed daily.

### Statistical analysis

Statistical analyses were performed using GraphPad Prism 9 software. Normality of the data was evaluated by the D'Agostino-Pearson test. One-way and two-way ANOVA with Tukey's multiple comparison test was used for the analysis of three groups. For paired or unpaired analysis of two groups, either Student's t test or Wilcoxon-Mann-Whitney test was used. Analyses are indicated in the figure legends. The data are reported as mean ± SD. Statistical significance was defined as p < 0.05.

## Author contributions

Conceptualization, V.V.O.; methodology, M.V.C., A.C., and V.V.O.; software, A.C.; validation, A.C. and M.V.C.; formal analysis, A.C. and M.V.C.; investigation, A.C., M.V.C., and F.E.v.d.H.; visualisation, M.V.C. and A.C.; resources, A.A.F.d.V. and V.V.O.; writing – original draft, M.V.C., A.C., C.L.M., and V.V.O.; writing – review & editing, M.V.C., A.C., S.A.J.L.O., C.L.M., and V.V.O.; supervision, S.A.J.L.O, C.L.M., and V.V.O.; project administration, V.V.O.; funding acquisition, A.C., S.A.J.L.O., C.L.M., and V.V.O.

## Conflict of interests

The authors declare no competing interests.

## Supplemental information

Document S1. Supplemental experimental procedures, Figures S1–S4, and Tables S1 and S2

Document S2. Article plus supplemental information
