# Supplementary material for: Engineered 3D vessel-on-chip using hiPSC-derived endothelial- and vascular smooth muscle cells
Source: Stem Cell Reports. 2021 Sep 2;16(9):2159–68. doi: 10.1016/j.stemcr.2021.08.003 (PMC8452600; doi:10.1016/j.stemcr.2021.08.003)
Supplement: Document S1. Supplemental experimental procedures, Figures S1–S4, and Tables S1 and S2 [file mmc1.pdf]

Stem Cell Reports, Volume *16*

Supplemental Information

Engineered 3D vessel-on-chip using hiPSC-derived endothelial- and vascular smooth muscle cells

Marc Vila Cuenca, Amy Cochrane, Francijna E. van den Hil, Antoine A.F. de Vries, Saskia A.J. Lesnik Oberstein, Christine L. Mummery, and Valeria V. Orlova

Inventory of Supplemental Information

Supplemental Figures and Legends:

Figure S1. Self-organization of VoC vascular network with hiPSC-ECs alone. Related to Figure 1.
Figure S2. Self-organization of VoC vascular network with prolonged culture. Related to Figure 1 & 2.
Figure S3. Characterization of hiPSC-VSMCs expressing GCaMP6f LV. Related to Figure 3.
Figure S4. Characterization of microvascular network upon EC-VSMC cross-talk modelling in VoC. Related to Figure 4.

Supplemental Tables:

Table S1. Flow experimental details.
Table S2. Complete list of hiPSC lines and batches used per experiment.

Supplemental Experimental Procedures

Supplemental References

Supplemental Videos:

Video S1. Perfusion of fluorescent beads (top panels) and FITC-Dextran (70 kDa)(bottom panels). Related to Figure 1.
Video S2. Intracellular Ca^{2+} Release in hiPSC-VSMC in VoC upon medium refreshment (left panel) and upon ET-I stimulation (right panel). Related to Figure 3.

Supplemental Figures and Legends

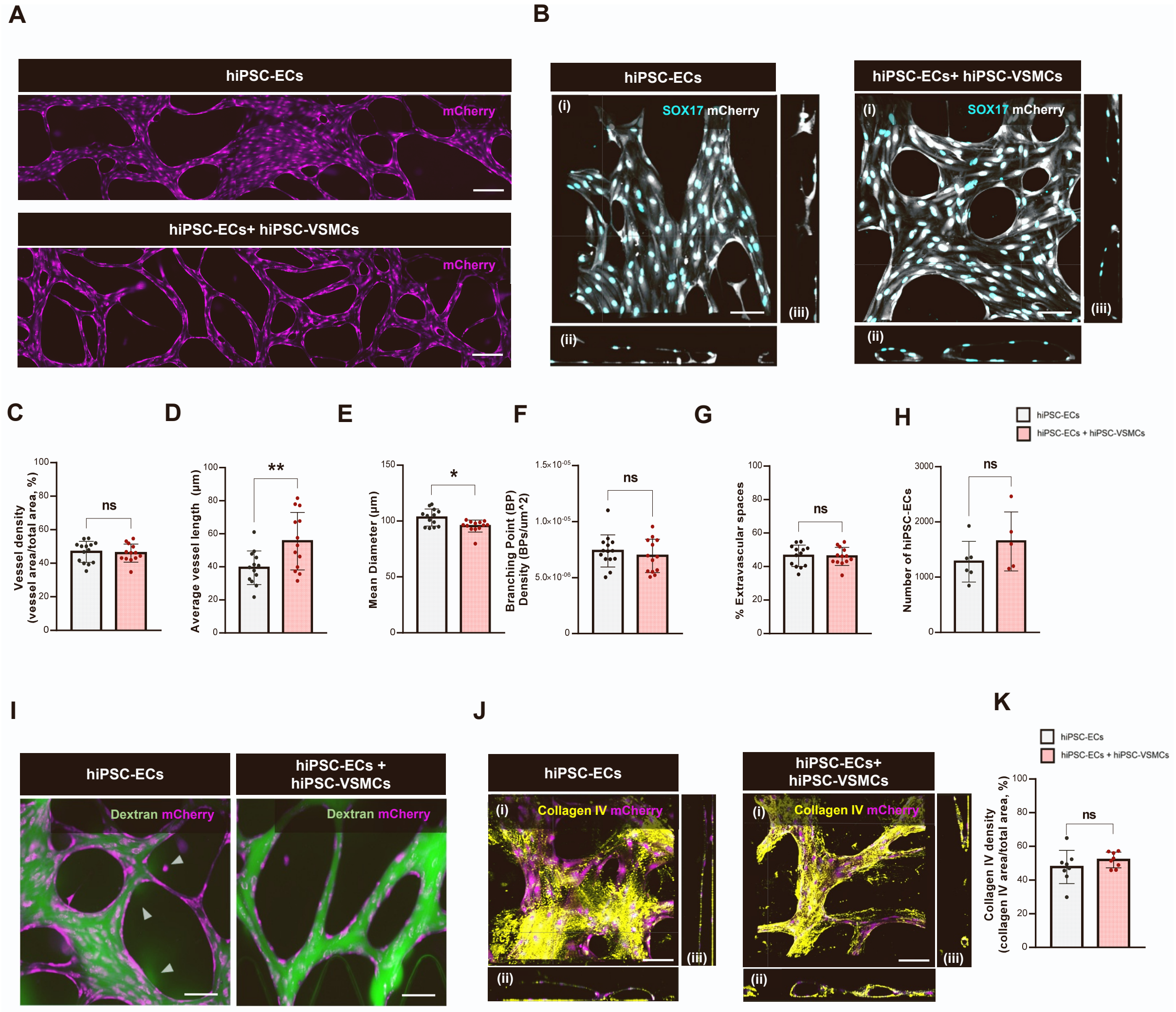

Figure S1. Self-organization of VoC vascular network with hiPSC-ECs alone. Related to Figure 1. (A) Representative immunofluorescence images of microvascular network showing hiPSC-ECs (magenta; mCherry). Images showing hiPSC-ECs cultured alone or with hiPSC-VSMCs respectively. 10x, scale bars 200 µm. (B) Representative confocal images of microvascular network showing hiPSC-ECs (grey; mCherry) and hiPSC-EC nuclei (cyan; SOX17). Images displaying xyz (i), xy (ii) and yz cross-sectional perspectives (iii). Images showing hiPSC-ECs cultured alone or with hiPSC-VSMCs respectively. 40x, scale bars 100 µm. (C-G) Quantification of vessel density (%, C), average vessel length (µm, D), mean diameter (µm, E), branching point (BP) density (BPs/µm2, F) and extravascular spaces (%, G) from hiPSC-ECs cultured alone or with hiPSC-VSMCs respectively are shown. Data are shown as ±SD from N=3, n=13; three independent experiments with three to six microfluidic channels per experiment. (H) Quantification of number of hiPSC-ECs from hiPSC-ECs cultured alone or with hiPSC-VSMCs respectively are shown. Data are shown as ±SD from N=3, n=6; three independent experiments with duplicates microfluidic channels per experiment. (I) Representative Immunofluorescence images showing hiPSC-ECs (magenta; mCherry) and perfusion of 70 kDa FITC-Dextran (green). Images showing hiPSC-ECs cultured alone or with hiPSC-VSMCs respectively. 10x, scale bars 100 µm. (J) Representative confocal images of microvascular network showing hiPSC-ECs (magenta; mCherry) and ECM (yellow; Collagen IV). Images displaying xyz (i), xy (ii) and yz cross-sectional perspectives (iii). Images showing hiPSC-ECs cultured alone or with hiPSC-VSMCs respectively. 40x, scale bars 100 µm. (K) Quantification of Collagen IV density (%) from hiPSC-ECs cultured alone or with hiPSC-VSMCs respectively are shown. Data are shown as ±SD from N=3, n=8; three independent experiments with two to three microfluidic channels per experiment. Wilcoxon-Mann-Whitney test. *$p < 0.05$, ** $p < 0.001$, ns = not significant.

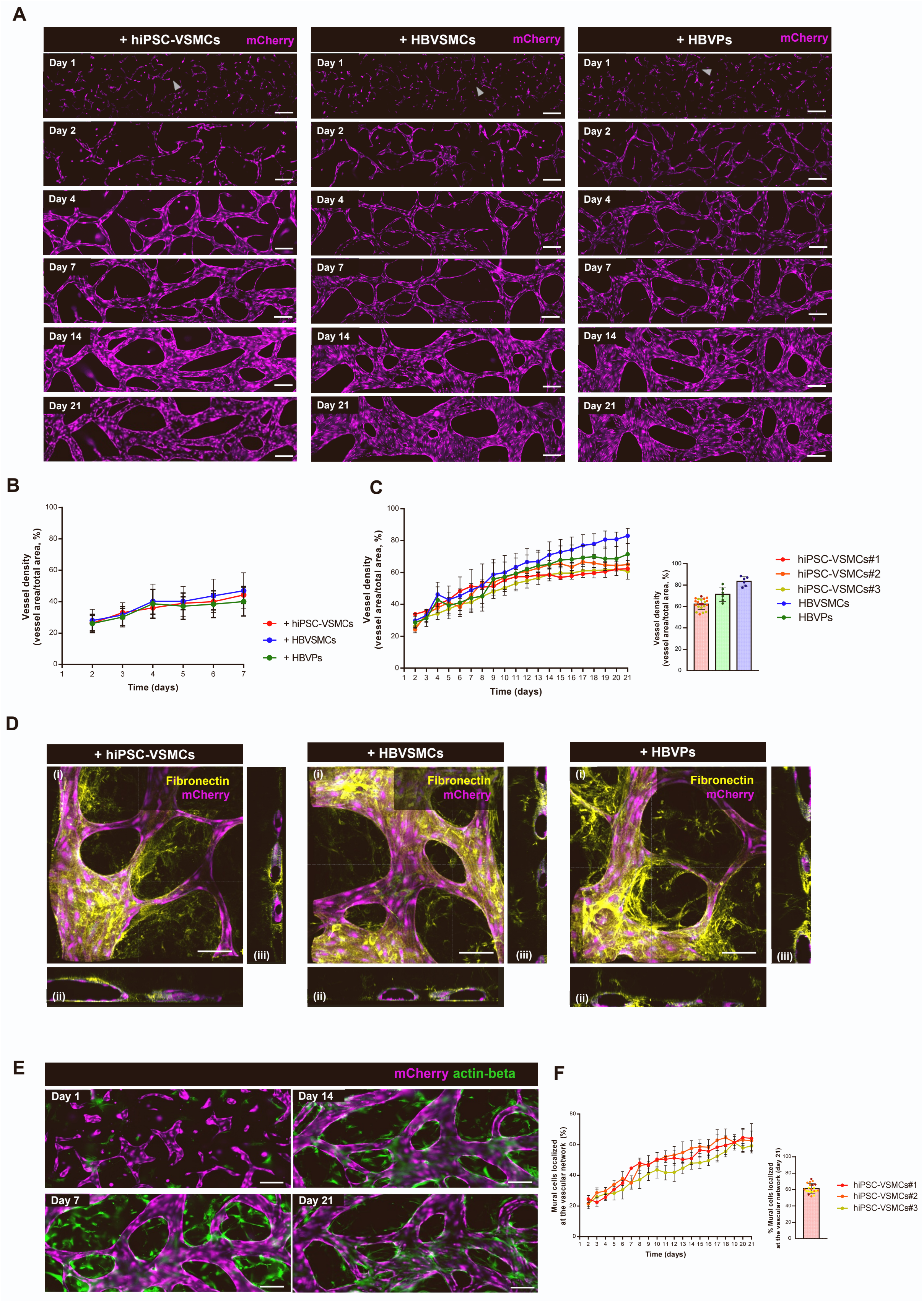

A
+ hiPSC-VSMCs mCherry
+ HBVSMCs mCherry
+ HBVPs mCherry
Day 1
Day 2
Day 4
Day 7
Day 14
Day 21
B
Vessel density (vessel area/total area, %)
Time (days)
+ hiPSC-VSMCs
+ HBVSMCs
+ HBVPs
C
Vessel density (vessel area/total area, %)
Time (days)
hiPSC-VSMCs#1
hiPSC-VSMCs#2
hiPSC-VSMCs#3
HBVSMCs
HBVPs
D
+ hiPSC-VSMCs
+ HBVSMCs
+ HBVPs
(i)
(ii)
(iii)
Fibronectin
mCherry
E
mCherry actin-beta
Day 1
Day 14
Day 7
Day 21
F
Mural cells localized at the vascular network (%)
Time (days)
% Mural cells localized at the vascular network (day 21)
hiPSC-VSMCs#1
hiPSC-VSMCs#2
hiPSC-VSMCs#3

Figure S2. Self-organization of VoC vascular network with prolonged culture. Related to Figure 1 & 2. (A) Daily immunofluorescence images (day 1, 2, 4, 7, 14, 21) showing hiPSC-ECs (magenta; mCherry) vascular cell self-organization, vasculogenesis and vascular remodelling over time. Images showing hiPSC-ECs cultured with hiPSC-VSMCs, HBVSMCs or HBVPs respectively. White arrowheads show vacuole formation at day 1. 10x, scale bars 200 µm. (B) Quantification of daily timepoints of the percentage of vessel density from day 2 (after formation of interconnected network) until day 7 with hiPSC-ECs cultured with hiPSC-VSMCs, HBVSMCs or HBVPs respectively. Data are shown as ±SD from N=3, n=20; three independent experiments with six to eight microfluidic channels per experiment. (C) Quantification of daily timepoints of the percentage of vessel density from day 2 (after formation of interconnected network) until day 21 with hiPSC-ECs cultured with hiPSC-VSMCs, HBVSMCs or HBVPs respectively. Quantification of vessel density at day 21 is shown as scatterplot with bar. Data are shown as ±SD from N=3, n=21; three independent experiments with three to nine microfluidic channels per experiment (hiPSC-VSMCs; AICS-0016 [#1-2] and LUMC0054iCTRL [#3]). N=1, n=6; one independent experiment with six microfluidic channels per experiment (HBVSMCs and HBVPs). (D) Representative confocal images of microvascular network showing hiPSC-ECs (magenta; mCherry) and ECM (yellow; Fibronectin). Images displaying xyz (i), xy (ii) and yz cross-sectional perspectives (iii). Images showing hiPSC-ECs cultured with hiPSC-VSMCs, HBVSMCs or HBVPs respectively. 40x, scale bars 100 µm. (E) Daily immunofluorescence images (day 1, 7, 14, 21) showing vascular cell self-organization and vascular remodelling over time. hiPSC-ECs (magenta; mCherry) and hiPSC-VSMCs (green; actin-beta). 20x, scale bar 200 µm. (F) Quantification of the percentage of mural cells associated with the hiPSC-EC lumen (% mural cells localized at the vascular network) from day 2 (after formation of interconnected network) until day 21. Quantification of the percentage of mural cells associated with the hiPSC-EC lumen at day 21 is shown as scatterplot with bar. Data are shown as ±SD from N=3, n=21; three independent experiments with three to nine microfluidic channels per experiment (hiPSC-VSMCs; AICS-0016 [#1-2] and LUMC0054iCTRL [#3]).

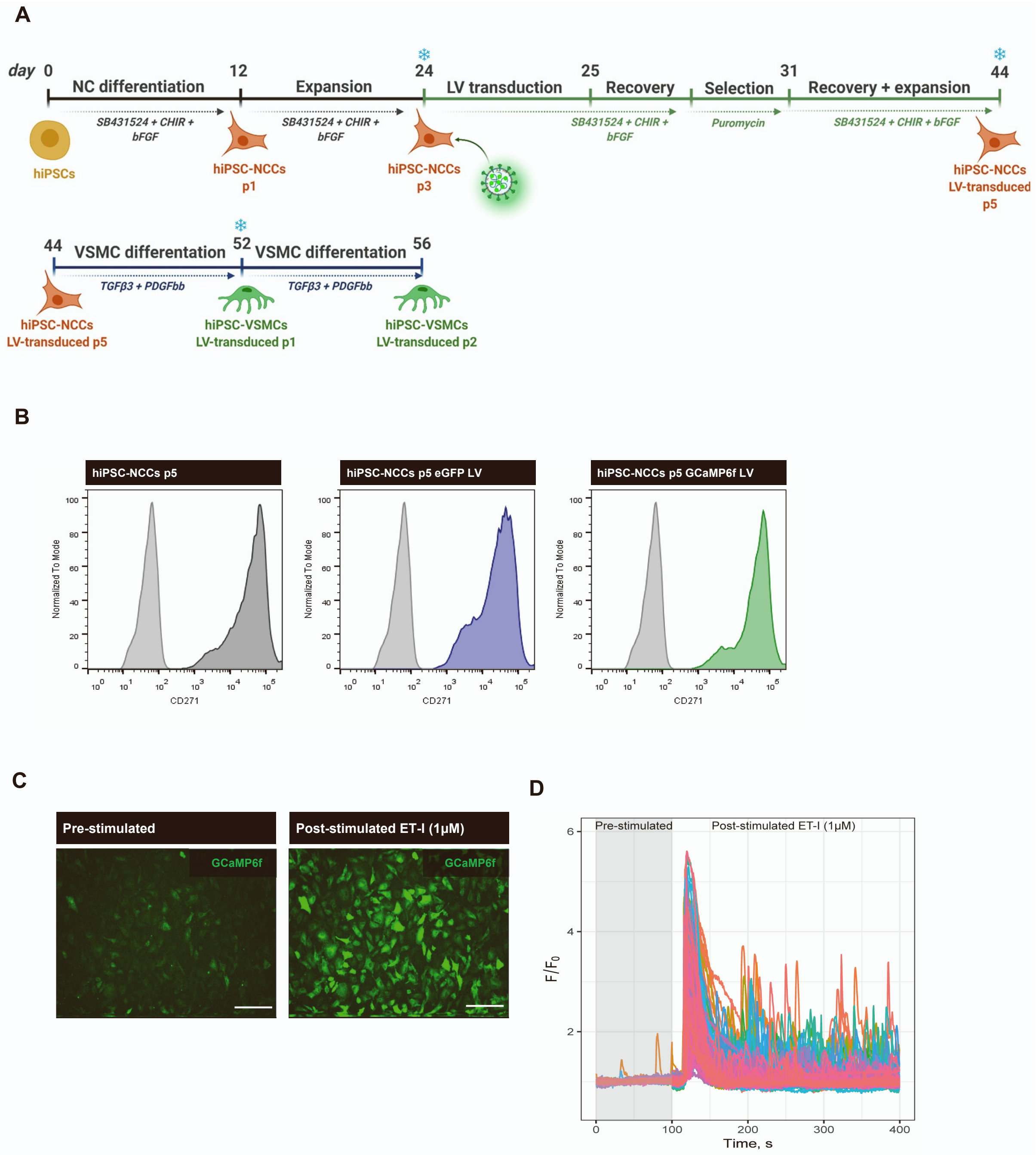

Figure S3. Characterization of hiPSC-VSMCs expressing GCaMP6f LV. Related to Figure 3. (A) Schematic illustration of the LV-transduction hiPSC-NC cells (hiPSC-NCCs) and hiPSC-VSMCs differentiation protocol. (B) FACS analysis of surface expression of CD271 in non-transduced hiPSC-NCCs (black filled histograms), hiPSC-NCCs expressing control LV (eGFP LV; blue filled histograms) or hiPSC-NCCs expressing GCaMP6f LV (GCaMP6f LV; green filled histograms). (C) Representative image from time-lapse of intracellular Ca^{2+} fluorescence in hiPSC-VSMCs (green; GCaMP6f) from in pre- and post- stimulated (ET-I 1 μM) states respectively. 10x, scale bar 200 μm. (D) Normalized average fluorescence intensity (F/F_0) within distinct region of interests (ROIs) over the time of the image sequence measured in hiPSC-VSMCs expressing GCaMP6f LV (one representative experiment). Each individual trace corresponds to one detected and tracked ROI. Time window marked in grey represents medium perfusion period (pre-stimulated state, 0–100 s). Post-stimulated state indicates a time of perfusion with ET-I (1 μM, 100-400 s).

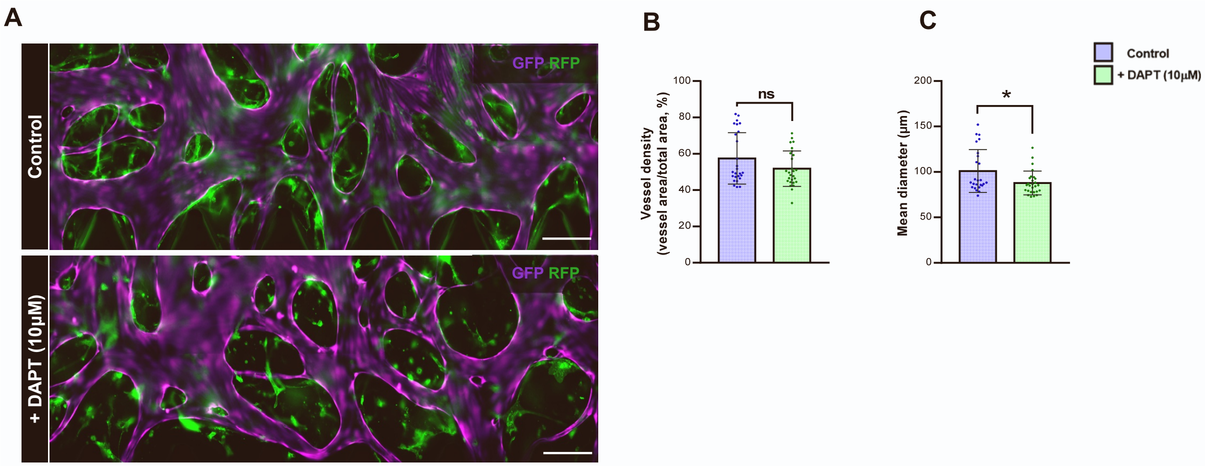

Figure S4. Characterization of microvascular network upon EC-VSMC cross-talk modelling in VoC. Related to Figure 4. (A) Representative immunofluorescence images of microvascular network showing hiPSC-ECs (magenta; GFP) and hiPSC-VSMCs (green; RFP) in control and DAPT (10 μM) supplemented conditions. 20x, scale bars 200 μm. (B-C) Quantification of the vessel density (%, B) and diameter (μm, C) of microfluidic channels in control and DAPT (10 μM) supplemented conditions at day 7. Data are shown as ±SD from N=3, n=27; three independent experiments with nine microfluidic channels per experiment. *$p < 0.05$, ns = not significant.

Supplemental Tables

Table S1. Flow experimental details.

Parameters	Values
Vessel length (µm)	597 – 934
Vessel diameter (µm)	75
Time for bead displacement (s)	4,5 – 20
Fluid proprieties (g/cm^3)	1
Wall shear stress (dyne/cm^2)	0,056 - 0,14
Flow velocity (mm/s)	0,05 – 0,13
Absolute Pressure difference (mBar)	0,5
Time flow convection stops (min)	14-18
Time flow is re-stablished (h)	24

Table S2. Complete list of hiPSC lines and batches used per experiment.

Figure	hiPSC-ECs		hiPSC-VSMCs	
	Line	Number of Batches	Line	Number of Batches
1F-K; 2C-E	LUMC0020iCTRL	1	LUMC0020iCTRL	1
	LUMC0054iCTRL	2	LUMC0054iCTRL	2
1M; 2G-I	LUMC0054iCTRL	2	LUMC0054iCTRL	3
	NCRM-1	1		
S1C-H, K	NCRM-1	3	AICS-0016	1
			LUMC0054iCTRL	2
S2B-C, F	NCRM-1	3 (+hiPSC-VSMCs); 1 (+ HBVPs/HBVSMCs)	LUMC0054iCTRL	1
			AICS-0016	2
3B, D-I	NCRM-1	3	LUMC0054iCTRL	3
4B-D, F-G; S4B-C	NCRM-1	3	AICS-0016	1
			AICS-0054	1
			LUMC0054iCTRL	1

Supplemental Experimental Procedures

hiPSC lines and maintenance

hiPSCs were maintained on recombinant vitronectin-coated plates in TeSR-E8, all from STEMCELL Technologies, according to the manufacturer's instructions. The following hiPSC lines were used: LUMC0054iCTRL (generated from kidney epithelial cells isolated from urine, http://hpscreg.eu/cell-line/LUMCi001-A) (Halaidych et al., 2018) and LUMC0020iCTRL (generated from skin fibroblasts, https://hpscreg.eu/cell-line/LUMCi028-A) (Zhang et al., 2014). NIH Center for Regenerative Medicine hiPSC line (NCRM-1, generated from CD34+ cord blood cells, https://hpscreg.eu/cell-line/CRMi003-A), obtained from RUDCR Infinite Biologics at Rutgers University, was modified in-house with a mCherry or GFP expression cassette under the human cytomegalovirus (hCMV) early enhancer/chicken β actin (CAG) promoter using a previously established protocol(Rostovskaya et al., 2012). The Allen Cell Collection lines: AICS-0016 (generated from skin fibroblasts, https://hpscreg.eu/cell-line/UCSFi001-A-3) with mEGFP insertion site at ACTB and AICS-0054 (generated from skin fibroblasts, https://hpscreg.eu/cell-line/UCSFi001-A-23) with mTagRFPT insertion site at AAVS1 were obtained from Coriell Institute for Medical Research.

Differentiation of hiPSCs towards ECs

hiPSCs were maintained in mTeSR-E8 and differentiated towards ECs using previously published protocols (Orlova et al., 2014a; Orlova et al., 2014b). For mesoderm induction (day 0-3), mTeSR-E8 medium was replaced with B(P)EL medium supplemented with 8 μM CHIR99021 (Tocris Bioscience, 4423). Cells were refreshed with vascular specification medium comprised of VEGF (50 ng/ml) and 10 µM SB431542 (Tocris Bioscience, 1614) in B(P)EL at day 3, day 6, and day 9. hiPSC-ECs were isolated on day 10 using CD31-Dynabeads™ (Thermo Fisher Scientific), as previously described (Orlova et al., 2014b; 2014a). hiPSC-ECs were expanded in complete EC growth medium comprised of Human Endothelial-serum free medium (EC-SFM) with 1% Human platelet poor serum (P2918, Sigma), VEGF (30 ng/ml) and bFGF (20 ng/ml), as described previously with minor modifications (Orlova et al., 2014a; Orlova et al., 2014b). hiPSC-ECs were expanded for additional 3-4 days post-isolation and cryopreserved using serum-free cryopreservation medium at passage number 1 (P1) (CryoStor™CS10) (StemCell Technologies, 07930).

Differentiation of hiPSCs towards VSMCs

hiPSC colonies were passaged and kept in hiPSC mTeSR-E8 and differentiated towards NCCs using previously published protocols (Halaidych et al., 2019a). After 2 days, the medium was changed to NC differentiation medium consisting of B(P)EL medium supplemented with 10 µM SB431542 (Tocris Bioscience, 1614), 1 µM CHIR99021 (Tocris Bioscience, 4423) and 10 ng/mL bFGF (Miltenyi Biotec, 130-093-842). Cells were refreshed every 2 days and kept in NC differentiation medium for 10-12 days. After 10-12 days NC cells (NCCs) were passaged with 1xTrypLE Select (Gibco, 12563029) and plated in 1:4 ratio on Matrigel-coated plates. hiPSC-NCCs were cryopreserved at passage number 3 (P3) using serum-free cryopreservation medium (CryoStor™CS10) (StemCell Technologies, 07930). NCCs were differentiated into VSMCs following a previously described protocol with minor modifications (Halaidych et al., 2019a). NCCs were plated at $3x10^4$ cells/cm^2 seeding density on 0.1% Gelatin (Sigma-Aldrich, G1890) coated plates in VSMC differentiation medium consisting of B(P)EL medium supplemented with 2 ng/mL TGF-β3 (PeproTech, 100-36E) and 10 ng/mL PDGF-BB (PeproTech, 100-14B). Cells were refreshed every 2 days and kept in VSMC differentiation medium for 8 days. Cells were passaged in a 1:4 splitting ratio at day 4. hiPSC-VSMCs were cryopreserved at passage number 1 (P1) using serum-free cryopreservation medium (CryoStor™CS10) (StemCell Technologies, 07930)

Primary mural cell culture

Human brain vascular pericytes (HBVPs) and Human brain vascular smooth muscle cells (HBVSMCs) were purchased from ScienceCell. HBVPs were cultured in Pericyte Medium (ScienceCell, 1201) supplemented with Pericyte Growth Supplement (ScienceCell, 1252) and 2% FBS. HBVSMCs were cultured in Smooth Muscle Cell Medium (ScienceCell, 1101) supplemented with Smooth Muscle Growth Supplement (ScienceCell, 1152) and 2% FBS. Cells were cryopreserved at passage number 3 (P3) using serum-free cryopreservation medium (CryoStor™CS10) (StemCell Technologies, 07930).

Cell preparation prior to VoC culture

hiPSC-ECs (P1) were thawed and cultured on gelatin-coated plates in complete EC growth medium composed of Human Endothelial-SFM (EC-SFM) with 1% platelet poor serum (PPS), VEGF (30 ng/ml) and bFGF (20 ng/ml), as described previously (Orlova et al., 2014b; 2014a) 4 days prior to VoC seeding.

hiPSC-VSMCs (P1) were thawed and cultured on gelatin-coated plates in B(P)EL medium supplemented with 2 ng/mL TGF-β3 (PeproTech, 100-36E) and 10 ng/mL PDGF-bb (PeproTech, 100-14B) 4 days prior to VoC seeding using previously described protocol with minor modifications (Protocol A, Halaidych et al., 2019). HBVPs (P3) were thawed and cultured in Pericyte Medium (ScienceCell, 1201) supplemented with Pericyte Growth Supplement (ScienceCell, 1252) and 2% FBS. HBVSMCs (P3) cultured in Smooth Muscle Cell Medium (ScienceCell, 1101) supplemented with Smooth Muscle Growth Supplement (ScienceCell, 1152) and 2% FBS. HBVPs and HBVSMCs were seeded on gelatin coated plates and cultured 4 days prior to VoC seeding.

Perfusion assessment in VoC system

For perfusion assessment in the VoC, hiPSC-ECs were labelled with 594-Agglutinin (1:600 in EGM-2, Vector laboratories) for 30 min in a CO_2 incubator to assess the vessel density after 7 days of culture. The chip was placed into the EVOS AUTO2 with an on-stage incubator for time-lapse image acquisition. First, basal fluorescence activity before the addition of fluorescent tracers was captured. Next, 70 µl of 70KDa FITC-Dextran (1:1000, Sigma) or 405-beads (1:10, Fluoro-Max Dyed Blue Aqueous Fluorescent Particles, B0200, ThermoFisher Scientific) in EGM-2 was added to one medium port and 50 µl of EGM-2 to all other media ports to induce interstitial gravity flow. Then, simultaneous image capturing at 20 fps using a 10x magnification objective was started. Confocal images were acquired to create a 3D stack using a DragonFly spinning disk (Andor) microscope with 20x magnification objective and processed using Imaris software (Bitplane, Oxford Instruments). Confocal images for dextran perfusion were acquired using a DragonFly spinning disk (Andor) microscope with 40x magnification objective and processed using Imaris 9.5 software (Bitplane, Oxford Instruments).

Flow parameters assessment in VoC system

To approximate the initial flow velocity in the microvessels, 405-beads (1:10, Fluoro-Max Dyed Blue Aqueous Fluorescent Particles, B0200, ThermoFisher Scientific) in PBS was added to the right media ports (100 µl) and to left connecting media ports (50 µl). Then, simultaneous image capturing with EVOSM7000 at 40 fps using a 4x magnification objective was started. Image stacks were imported and processed using Imaris 9.5 software (Bitplane, Oxford Instruments). 405-beads were tracked by using the 3D rendering and cell-tracking function over time. Three vascular segments were selected as ROI to obtain the track displacement length and duration of the beads. Time until convection stops was estimated when the velocity of the 405-beads in the media channel was 0. Wall shear stress, pressure and flow velocity were calculated with https://darwin-microfluidics.com/blogs/tools/microfluidic-flowrate-and-shear-stress-calculator.

Immunostaining and Microscopy

Cells in VoCs were fixed *in situ* in 4% paraformaldehyde (PFA) for 30 min at RT. Cell plasma membranes were permeabilized with 0.5% Triton X-100 for 15 min at RT and washed 3 times for 10 mins between each step with PBS, then blocking buffer (2% BSA) was added for 3 hours at RT. Primary antibodies (1:200 volume ratio in 1% BSA), against CD31 (PECAM1, Mouse M0823 DAKO), SOX17 (goat, AF1924, R&D Systems), SM22 (TAGLIN; Rabbit, ab14106, Abcam), Collagen IV (goat, AB769, Sigma), Fibronectin (Rabbit, F3648, Sigma) used to identify hiPSC-ECs, hiPSC-EC nuclei, mural cells and ECM respectively, were incubated overnight at 4 ºC. Secondary antibodies (1:300 volume ratio in 1% BSA), were incubated for 2 hours at RT after 3 times 15 min PBS washes. VoCs were imaged using EVOS M7000 using 10x magnification objective. A customised plate layout that allowed for automated imaging and stitching to produce images of complete microfluidic channel for all fluorescent channels was used. For 3D stacks, images were taken using a DragonFly spinning disk (Andor) microscope with 40x magnification objective and post-processing performed and processed using Imaris 9.5 software (Bitplane, Oxford Instruments).

Characterisation of vascular and perivascular parameters in 2D

Images from the whole microfluidic channel (acquired using EVOS) were quantified using pipelines developed on the free open source CellProfiler software (https://cellprofiler.org/) (Carpenter et al., 2006). Briefly, for EC nuclei number, pre-processing steps were applied to all images to enhance image features and a gaussian filter to reduce unspecific object identification. A Gaussian filter applied to mural cell images before object identification was used to measure object morphology and staining intensity (GCaMP6f). Two filter steps were applied to images of vascular network to reduce non-specific segmentation from cell junctions and a minimum cross-entropy thresholding method was used to produce a binarized image. For ECM area quantification, two filter steps were applied to images of Collagen IV and robust background thresholding method was used to produce a binarized image. The

binarized images from the CellProfiler output were then analyzed using the freely available ImageJ software with the plugin (https://imagej.nih.gov/ij/ ,https://imagej.net/DiameterJ) (Hotaling et al., 2015)..

Characterization of mural cell parameters in 3D
For 3D quantitative analysis, surface-rendering of individual mural cells (SM22) was performed and processed using Imaris 9.5 software (Bitplane, Oxford Instruments). Mean object SM22 intensity and number of SM22 positive objects was obtained from each 3D stack. To define the mural cells in contact with hiPSC-ECs, surface-rendering of vessel structure (CD31, mCherry and GFP) was performed and distance of SM22 objects was defined as 0 µm (contact) and >0 µm (no contact) from the object hiPSC-ECs. SM22 objects where filtered based on distance and mean object SM22 intensity was obtained.

Plasmid constructs
The lentiviral vector (LV) shuttle plasmid pLV.hCMV-IE.GCaMP6f.IRES.PurR.hHBVPRE was generated in a multistep procedure using pGP-CMV-GCaMP6f (Addgene, Watertown, MA; plasmid number 40755) and pLV.hCMV-IE.IRES.PurR.hHBVPRE as starting constructs (Neshati et al., 2014). pLV.hCMV-IE.GCaMP6f.IRES.PurR.hHBVPRE contains a human cytomegalovirus immediate-early gene (hCMV-IE) promoter driving expression of a bicistronic mRNA encoding the ultra-sensitive $[Ca^{2+}]_{cyt}$ sensor GCaMP6f (Chen et al., 2013) and *Streptomyces alboniger* puromycin-N-acetyltransferase. The LV shuttle plasmid pLV.hCMV-IE.eGFP.PurR.hHBVPRE was generated by insertion of the *Aequorea victoria* enhanced green fluorescent protein (eGFP)-encoding 754-bp SmaI×EcoRI fragment of pEGFP (Clontech - Takara Bio Europe, Saint-Germain-en-Laye, France) behind the hCMV-IE promoter of pLV.hCMV-IE.IRES.PurR.hHBVPRE (Neshati et al., 2014). To this end, the insert was combined with the 8122-bp SmaI×EcoRI fragment of pLV.hCMV-IE.IRES.PurR.hHBVPRE (Neshati et al., 2014). Recombinant plasmid construction was done with enzymes from New England Biolabs (Bioké, Leiden, the Netherlands) or Fermentas (ThermoFisher Scientific) using standard procedures or following the instructions provided with specific reagents. The plasmids were amplified in *Escherichia coli* GeneHogs (ThermoFisher Scientific) cells and purified using LabNed Plasmid Maxiprep Kits (ITK diagnostics, Uithoorn, the Netherlands).

LV production
LV particles were produced essentially as described previously (Liu et al., 2018) except that PEI MAX 40K (Polysciences Europe, Hirschberg an der Bergstraße, Germany) instead of PEI 25K was used as transfection agent and the polyethyleneimine-DNA complexes were left on the cells for only 4 hours.

LV transduction of hiPSC-VSMCs
The LV shuttle plasmid pLV.hCMV.-IE.GCaMP6f(+).IRES.PurR.hHBVPRE was used to express GCaMP6f in LUMC0054iCTRL hiPSC-NCCs P3. The LV shuttle plasmid pLV.hCMV.-IE.eGFP.IRES.PurR.hHBVPRE) was used to express pEGFP in LUMC0054iCTRL hiPSC-NCCs P3. Briefly, one day after seeding 40.000 cells/12-well on Matrigel-coated plates, hiPSC-NCCs were transduced with 2.5 µl viral particles in B(P)EL medium overnight. 96h post-transduction with complete NC differentiation medium infected cells were selected with 1 µg/mL puromycin (Sigma, P7255). After 4 days, remaining cells were expanded (1:3 ratio) Matrigel-coated plates then dissociated with 1xTrypLE Select and cryopreserved at passage number 3 (P3) using serum-free cryopreservation medium (CryoStor™CS10) (StemCell Technologies, 07930). Next, hiPSC-NC cells (hiPSC-NCCs) were differentiated into hiPSC-VSMCs as described previously with minor modifications (Halaidych et al., 2019a).

Flow cytometry analysis
hiPSC-NCCs were dissociated with 1xTrypLE Select and washed once with FACs buffer containing 10% FBS, and once with FACs buffer. CD271-BV421 (BD Biosciences, 562562, 1:100) surface antibody was used for the FACS staining. Analysis of samples was performed on the MACSQuant VYB (Miltenyi Biotec, 130-096-116).

Assessment of intracellular Ca^{2+} release in hiPSC-VSMCs
Intracellular Ca^{2+} release in hiPSC-VSMCs engineered to express GCaMP6f was performed as described previously (Halaidych et al., 2019a). Briefly, cells were seeded into a microfluidic chip (Vena8 Endothelial+, Cellix Ltd) coated with 50 µg/mL bovine fibronectin (Sigma) at density 10^4 cells/µL. Cells were kept in a CO_2 incubator for 3 hours before functional analysis. Single channel perfusion was enabled by a PC-controlled syringe pump (Mirus Evo Nanopump, Cellix). The microfluidic chip was placed into a live imaging chamber (+37°C, 5% CO2, humidified) mounted on a Leica AF6000

microscope. Image sequences of fluorescence were captured at 2 frames per second using a 10x magnification objective with 2x2 binning (spatial resolution: 2.28 µm/pix). First, basal fluorescence activity upon B(P)EL medium flow was captured. Then image capturing was paused and the inlet reservoir was filled with 1 µM Endothelin-I (Sigma) diluted in B(P)EL medium. Flow was applied again and simultaneous image capturing was continued.

Assessment of intracellular Ca^{2+} release in the VoC

Intracellular Ca^{2+} release upon medium refreshment and upon stimulation with the vasoconstrictor (ET-I) was analyzed on day 7 of VoC culture. Sequences of images prior to- (basal state) and after medium refreshment were captured using EVOS M7000 with a 10x objective. For the medium refreshment, medium from all ports of the microfluidic channel was first removed and gravity-driven flow was induced by the addition of 100 µl medium to the right media ports and 50 µl medium to left connecting media ports. After 30s, fluorescence activity of the whole microfluidic channel was captured. For real-time intracellular Ca^{2+} release upon stimulation with the vasoconstrictor, the microfluidic chip was placed into a humidified live cell imaging chamber (+37°C, 5% CO_2) and mounted on a DragonFly spinning disk microscope (Andor) with a 20x magnification objective on day 7 of culture. First, medium from all ports was removed and refreshed with 30 µl of EGM-2. After 30 min, basal fluorescence activity was captured for 5 seconds. Next, gravity-driven flow was induced by the addition of 60 µl EGM-2 or EGM-2 supplemented with 1.5 µM ET-I (Sigma) with a final concentration of 1 µM after the addition to the right medium ports containing 30 µl of EGM-2. Then, simultaneous image capturing was continued for 160 seconds. Image sequences of fluorescence were captured at 4 frames per second. After simultaneous image capturing, confocal images were acquired to create a 3D stack and processed using Imaris 9.5 software (Bitplane, Oxford Instruments).

Analysis of intracellular Ca^{2+} Release

Images sequences were processed using a freely available plugin ''LC Pro'' for ImageJ (https://imagej.nih.gov/ij/plugins/lc-pro/index.html) (Yip and Sham, 2012). Free open-source CellProfiler software (https://cellprofiler.org/) (Carpenter et al., 2006) was used to determine the total number of cells in a field of view. Output data were analysed as previously described (Halaidych et al., 2019a; Halaidych et al., 2019b).

Supplemental References

Carpenter, A.E., Jones, T.R., Lamprecht, M.R., Clarke, C., Kang, I.H., Friman, O., Guertin, D.A., Chang, J.H., Lindquist, R.A., Moffat, J.*, et al.* (2006). CellProfiler: image analysis software for identifying and quantifying cell phenotypes. Genome Biol *7*, R100.

Chen, T.W., Wardill, T.J., Sun, Y., Pulver, S.R., Renninger, S.L., Baohan, A., Schreiter, E.R., Kerr, R.A., Orger, M.B., Jayaraman, V.*, et al.* (2013). Ultrasensitive fluorescent proteins for imaging neuronal activity. Nature *499*, 295-300.

Halaidych, O.V., Cochrane, A., van den Hil, F.E., Mummery, C.L., and Orlova, V.V. (2019a). Quantitative Analysis of Intracellular Ca(2+) Release and Contraction in hiPSC-Derived Vascular Smooth Muscle Cells. Stem Cell Reports *12*, 647-656.

Halaidych, O.V., Freund, C., van den Hil, F., Salvatori, D.C.F., Riminucci, M., Mummery, C.L., and Orlova, V.V. (2018). Inflammatory Responses and Barrier Function of Endothelial Cells Derived from Human Induced Pluripotent Stem Cells. Stem Cell Reports *10*, 1642-1656.

Halaidych, O.V., Mummery, C.L., and Orlova, V.V. (2019b). Quantifying Ca(2+) signaling and contraction in vascular pericytes and smooth muscle cells. Biochem Biophys Res Commun *513*, 112-118.

Hotaling, N.A., Bharti, K., Kriel, H., and Simon, C.G., Jr. (2015). DiameterJ: A validated open source nanofiber diameter measurement tool. Biomaterials *61*, 327-338.

Liu, J., Volkers, L., Jangsangthong, W., Bart, C.I., Engels, M.C., Zhou, G., Schalij, M.J., Ypey, D.L., Pijnappels, D.A., and de Vries, A.A.F. (2018). Generation and primary characterization of iAM-1, a versatile new line of conditionally immortalized atrial myocytes with preserved cardiomyogenic differentiation capacity. Cardiovasc Res *114*, 1848-1859.

Neshati, Z., Liu, J., Zhou, G., Schalij, M.J., and de Vries, A.A. (2014). Development of a lentivirus vector-based assay for non-destructive monitoring of cell fusion activity. PLoS One *9*, e102433.

Orlova, V.V., Drabsch, Y., Freund, C., Petrus-Reurer, S., van den Hil, F.E., Muenthaisong, S., Dijke, P.T., and Mummery, C.L. (2014a). Functionality of endothelial cells and pericytes from human pluripotent stem cells demonstrated in cultured vascular plexus and zebrafish xenografts. Arterioscler Thromb Vasc Biol *34*, 177-186.

Orlova, V.V., van den Hil, F.E., Petrus-Reurer, S., Drabsch, Y., Ten Dijke, P., and Mummery, C.L. (2014b). Generation, expansion and functional analysis of endothelial cells and pericytes derived from human pluripotent stem cells. Nat Protoc *9*, 1514-1531.

Rostovskaya, M., Fu, J., Obst, M., Baer, I., Weidlich, S., Wang, H., Smith, A.J., Anastassiadis, K., and Stewart, A.F. (2012). Transposon-mediated BAC transgenesis in human ES cells. Nucleic Acids Res *40*, e150.

Yip, K.P., and Sham, J.S. (2012). Tracking stars: automated two-dimensional analysis of Ca(2)(+) events. Focus on "Automated region of interest analysis of dynamic Ca(2)(+) signals in image sequences". Am J Physiol Cell Physiol *303*, C233-235.

Zhang, M., D'Aniello, C., Verkerk, A.O., Wrobel, E., Frank, S., Ward-van Oostwaard, D., Piccini, I., Freund, C., Rao, J., Seebohm, G*., et al.* (2014). Recessive cardiac phenotypes in induced pluripotent stem cell models of Jervell and Lange-Nielsen syndrome: disease mechanisms and pharmacological rescue. Proc Natl Acad Sci U S A *111*, E5383-5392.
