# Supplementary material for: Engineered 3D vessel-on-chip using hiPSC-derived endothelial- and vascular smooth muscle cells
Source: Stem Cell Reports. 2021 Sep 2;16(9):2159–68. doi: 10.1016/j.stemcr.2021.08.003 (PMC8452600; doi:10.1016/j.stemcr.2021.08.003)
Supplement: Document S2. Article plus supplemental information [file mmc4.pdf]

Stem Cell Reports
Report
OPEN ACCESS

Engineered 3D vessel-on-chip using hiPSC-derived endothelial- and vascular smooth muscle cells

Marc Vila Cuenca,[1,2,4] Amy Cochrane,[1,4] Francijna E. van den Hil,[1] Antoine A.F. de Vries,[3] Saskia A.J. Lesnik Oberstein,[2] Christine L. Mummery,[1] and Valeria V. Orlova[1,*]
[1]Department of Anatomy and Embryology, Leiden University Medical Center, Einthovenweg 20, 2333ZA Leiden, the Netherlands
[2]Department of Clinical Genetics, Leiden University Medical Center, 2333ZA Leiden, the Netherlands
[3]Department of Cardiology, Leiden University Medical Center, 2333ZA Leiden, the Netherlands
[4]These authors contributed equally
*Correspondence: v.orlova@lumc.nl
https://doi.org/10.1016/j.stemcr.2021.08.003

SUMMARY

Crosstalk between endothelial cells (ECs) and pericytes or vascular smooth muscle cells (VSMCs) is essential for the proper functioning of blood vessels. This balance is disrupted in several vascular diseases but there are few experimental models which recapitulate this vascular cell dialogue in humans. Here, we developed a robust multi-cell type 3D vessel-on-chip (VoC) model based entirely on human induced pluripotent stem cells (hiPSCs). Within a fibrin hydrogel microenvironment, the hiPSC-derived vascular cells self-organized to form stable microvascular networks reproducibly, in which the vessels were lumenized and functional, responding as expected to vasoactive stimulation. Vascular organization and intracellular Ca^{2+} release kinetics in VSMCs could be quantified using automated image analysis based on open-source software CellProfiler and ImageJ on widefield or confocal images, setting the stage for use of the platform to study vascular (patho)physiology and therapy.

INTRODUCTION

Crosstalk between endothelial cells (ECs) and mural cells (pericytes and vascular smooth muscle cells [VSMCs]) is pivotal for proper function of many blood vessels. Aberrant EC-mural cell crosstalk often leads to vascular diseases that range from hypertension, atherosclerosis, vascular calcification, and coronary artery disease to stroke and other conditions (Owens et al., 2004). Animal models, including genetically modified mice, are widely used to study vascular development and disease, but these do not always capture patient phenotypes unless the mutations are homozygous deletions, and differences associated with the genetic background and susceptibility are not evident in mice (Berry et al., 2019; Van Norman, 2020). Human induced pluripotent stem cells (hiPSCs) generated from heathy individuals and patients are a useful source of vascular cells and they do reflect the genetic background of the individual from whom they are derived (Samuel et al., 2015). Several methods have been described to generate ECs and VSMCs from hiPSCs and some have already been used to model vascular disease-specific abnormalities (Cochrane et al., 2019).

Nevertheless, and despite recent advances, many current *in vitro* models of blood vessels fail to emulate the integrated, complex and multicell-type composition of the human vasculature and do not include a mimic of blood flow (Duval et al., 2017). To address this, microfluidic devices have been engineered that do incorporate these features and provide the environment for the formation of multi-cell type 3D tissues and vessels-on-chip (VoC) (Tronolone and Jain, 2021). Typically, cells incorporated in these microphysiological devices are derived from non-human sources, human (tumor) cell lines, or directly from primary human tissue. Primary human cells provide the closest mimic to human blood vessels but are of limited availability and of variable genetic origin (Tronolone and Jain, 2021). While hiPSC derivatives are now regarded as an alternative, they have so far largely been used in combination with primary cells in microfluidic chips. For example, human primary ECs, or hiPSC-ECs have been combined with human primary mural cells (Campisi et al., 2018; van Dijk et al., 2020; van Duinen et al., 2019) but not with mural cells derived from hiPSC, precluding opportunities to replicate (patient-specific) vascular diseases originating in the mural cells.

Here, we overcome these limitations by incorporating hiPSC-ECs with hiPSC-VSMCs in entirely hiPSC-based VoCs. We have previously described robust protocols to derive ECs and VSMCs from multiple healthy hiPSC lines with little batch-to-batch variability (Halaidych et al., 2018, 2019; Orlova et al., 2014a, 2014b). The functionality of hiPSC-VoC was compared with similar VoCs containing hiPSC-ECs and human primary mural cells of the same development origin, namely human brain vascular smooth muscle cells (HBVSMCs) and primary human brain vascular pericytes (HBVPs). In all cases, we showed the microenvironment in the microfluidic device supported the formation of a 3D perfusable, self-assembled microvascular network. We optimised the culture conditions and developed an automated quantification

This is an open access article under the CC BY license (http://creativecommons.org/licenses/by/4.0/).

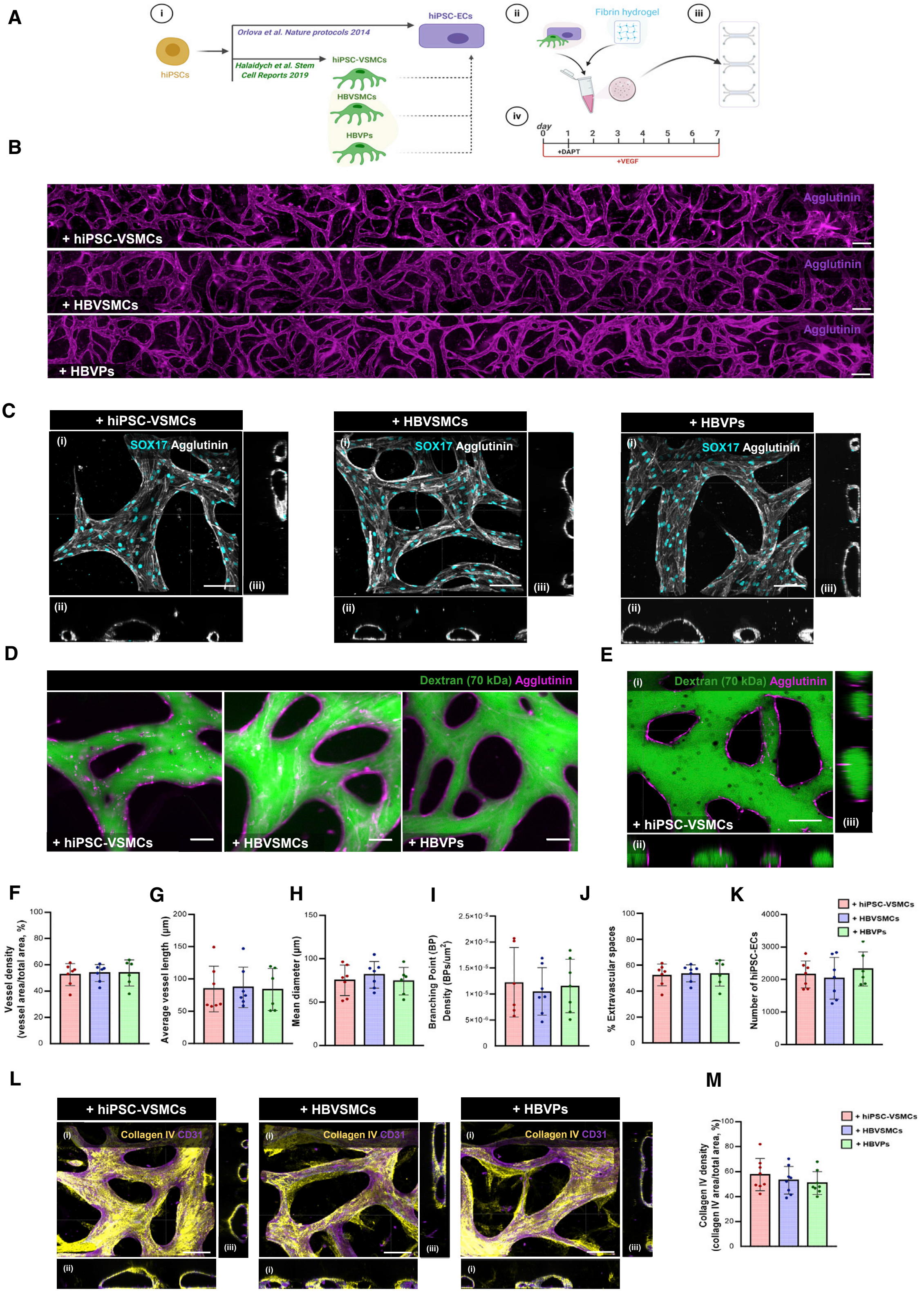

(legend on next page)

framework of the vascular network, mural cell morphology, EC-mural cell interaction and extracellular matrix (ECM) composition. Finally, we demonstrated a functional application: the automated quantification of vasoactive responses.

RESULTS

Characterization of the VoC model

Using the commercially available AIM Biotech 3D cell culture chips, hiPSC-ECs (Halaidych et al., 2018; Orlova et al., 2014a, 2014b) were combined with hiPSC-VSMCs (Halaidych et al., 2019), HBVSMCs or HBVPs (Figure 1Ai) in a fibrin hydrogel (Figure 1Aii) and the cell/gel mix was injected into the middle channel of the microfluidic chip (Figure 1Aiii). As a control, hiPSC-ECs in a fibrin hydrogel without mural cells were used (Figure S1). Endothelial growth medium-2 (EGM-2), supplemented with vascular endothelial growth factor (VEGF) (50 ng/mL), was used to support microvascular network formation. Microfluidic chips were perfused through gravity-driven flow by adding 100 μL of medium to the inlet and 50 μL to the outlet of each medium channel. The gravity-driven flow was re-established every 24 h allowing medium exchange in the microfluidic chip (Table S1). On day 1, after hiPSC-ECs had begun to self-organize, γ-secretase inhibitor DAPT (10 μM) was added to the medium for 24 h to promote hiPSC-EC sprouting (Figure 1Aiv). Vacuoles started to appear in hiPSC-ECs 12–24 h after seeding (Figure S2A, white arrowheads) followed by proliferation and remodeling up to 72 h. An interconnected microvascular network formed as early as day 2 (Figure S2A) and this spanned the complete microfluidic channel by day 7 (Figures 1B, S1A, and S2A). All combinations of mural cells with hiPSC-ECs resulted in vascular lumen formation (Figure 1C). Furthermore, these lumenized networks were perfusable by fluorescent beads (10 μm) or FITC-Dextran (70 kDa) (Figures 1D and 1E; Video S1) under gravity-driven flow. Microvascular networks formed in the presence of hiPSC-VSMCs or primary mural cells showed similar morphologies, with no significant difference in vessel density (%, Figure 1F), average vessel length (μm, Figure 1G), mean vessel diameter (μm, Figure 1H), branching point (BP) density (BP/μm^2, Figure 1I), extravascular spaces (%, Figure 1J), or number of hiPSC-ECs (Figure 1K). In contrast, microvascular networks formed by hiPSC-ECs alone were less organized than microvascular networks with hiPSC-VSMCs (Figure S1A). Although lumen formation was observed in microvascular networks formed using only hiPSC-ECs, these appeared irregular and broken (Figure S1B). The instability of microvascular networks without hiPSC-VSMCs was also evidenced by increased leakage of FITC-Dextran from the vessel network (70 kDa; Figure S1I). There were, however, no significant differences in vessel density (%, Figure S1C), branching point (BP)

Figure 1. Characterization of VoC

(A) Schematic of the VoC protocol. hiPSC-ECs were cultured with hiPSC-VSMCs, HBVSMCs, or HBVPs (i). Cells were mixed in a fibrin hydrogel (ii) and injected into (AIM Biotech) microfluidic chips (iii). EGM-2 was supplemented with VEGF (50 ng/mL), chips were refreshed daily for 7 days. EGM-2 was also supplemented with DAPT (10 μM) on day 1 for 24 h (iv).

(B) Representative immunofluorescence images of microvascular network showing hiPSC-EC (magenta; agglutinin) vessels spanning the complete length of the microfluidic channel. Images showing hiPSC-ECs cultured with hiPSC-VSMCs, HBVSMCs, or HBVPs, respectively (10×). Scale bars, 200 μm.

(C) Representative confocal images of microvascular network showing hiPSC-ECs (gray; agglutinin) and hiPSC-EC nuclei (cyan; SOX17). Images displaying xyz (i), xy (ii), and yz cross-sectional perspectives (iii). Images showing hiPSC-ECs cultured with hiPSC-VSMCs, HBVSMCs, or HBVPs, respectively (40×). Scale bars, 100 μm.

(D) Representative Immunofluorescence images showing hiPSC-ECs (magenta; agglutinin) and perfusion of 70 kDa FITC-Dextran (green). Images showing hiPSC-ECs cultured with hiPSC-VSMCs, HBVSMCs, or HBVPs, respectively (10×). Scale bars, 50 μm.

(E) Representative confocal images showing hiPSC-ECs (magenta; agglutinin) and perfusion of 70 kDa FITC-Dextran (green) in hiPSC-VoC. Images displaying xyz (i), xy (ii), and yz cross-sectional perspectives (iii) (40×). Scale bars, 100 μm.

(F–K) Quantification of vessel density (%) (F), average vessel length (μm) (G), mean diameter (μm) (H), branching point (BP) density (BPs/μm^2) (I) extravascular spaces (%) (J), and number of hiPSC-ECs (K), from hiPSC-ECs cultured with hiPSC-VSMCs, HBVSMCs, or HBVPs, respectively, are shown. Data are shown as ±SD from N = 3, n = 6; three independent experiments with two microfluidic channels per experiment.

(L) Representative confocal images of microvascular network showing hiPSC-ECs (magenta; CD31) and ECM (yellow; collagen IV). Images displaying xyz (i), xy (ii), and yz cross-sectional perspectives (iii). Images showing hiPSC-ECs cultured with hiPSC-VSMCs, HBVSMCs, or HBVPs, respectively (40×). Scale bars, 100 μm.

(M) Quantification of collagen IV density (%) from hiPSC-ECs cultured with hiPSC-VSMCs, HBVSMCs, or HBVPs, respectively, are shown. Data are shown as ±SD from N = 3, n = 8; three independent biological replicates with two to three microfluidic channels per experiment. One-way ANOVA with Tukey's multiple comparison.

See also Figures S1 and S2A–S2D and Video S1.

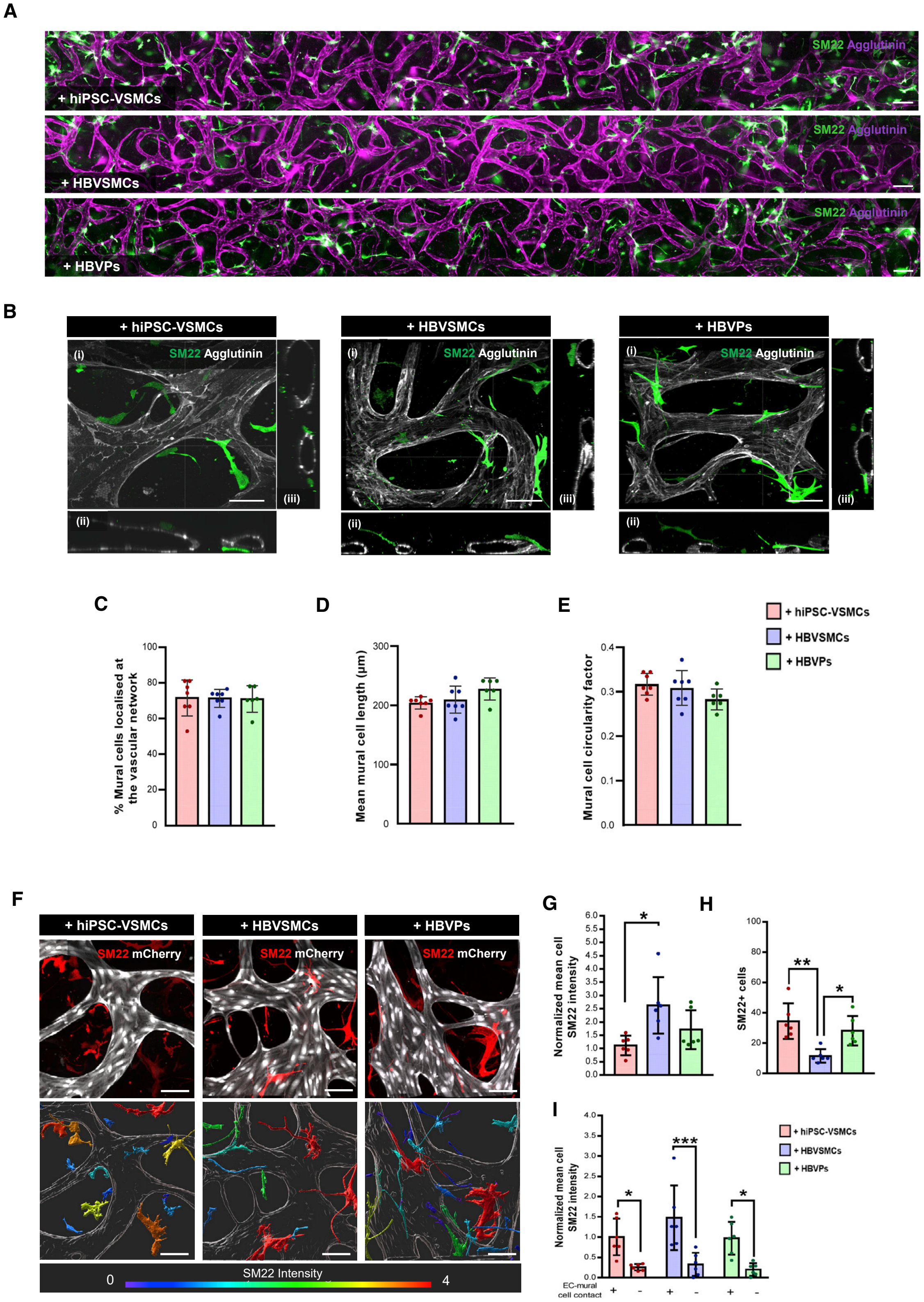

(legend on next page)

density (BP/μm^2, Figure S1F), extravascular space (%, Figure S1G), or number of hiPSC-ECs (Figure S1H) in microvascular networks formed either with or without hiPSC-VSMCs. By contrast, average vessel length (μm, Figure S1D) and mean vessel diameter (μm, Figure S1E) were significantly reduced or increased respectively in microvascular networks formed from hiPSC-ECs alone compared with microvascular networks formed in the presence of hiPSC-VSMCs. No significant differences were found in ECM deposition, evidenced by changes in the relative density of collagen IV, between any cell combinations (Figures 1L, 1M, S1J, and S1K). Furthermore, the presence of fibronectin was confirmed by immunostaining in microvascular networks formed with mural cells (Figure S2D). Finally, long-term culture of hiPSC-ECs in VoC with mural cells demonstrated that the microvascular network architecture was stable and underwent continuous increase in vessel density over 21 days (Figures S2A–S2C), although we observed an increase in density of vessel networks formed with primary HBVSMCs on day 21 (Figures S2A–S2C).

Characterization of hiPSC-VSMCs and primary mural cells in the VoC model

hiPSC-VSMCs, HBVSMCs, and HBVPs self-organized and self-oriented toward the developing hiPSC-EC microvascular networks, as early as day 1 (Figure S2E). On day 7, all mural cells were located at extravascular positions along the entire length of the microvascular network (Figure 2A), surrounding hiPSC-EC lumens (Figure 2B). No significant difference was observed in the percentage of hiPSC-VSMCs, HBVSMCs, or HBVPs associated with the hiPSC-EC lumen (% mural cells localized at the vascular network, Figure 2C). Quantification of mural cell morphology also showed no significant difference in the cell length (μm, Figure 2D) or cell circularity (Figure 2E). Analysis of the contractile marker SM22 in mural cells in microvascular networks showed a significantly lower normalized mean cell intensity in hiPSC-VSMCs compared with HBVSMCs (Figures 2F and 2G) while the total number of SM22 + cells was significantly lower in microvascular networks formed with HBVSMCs (Figure 2H). Notably, we also observed that all mural cells displayed significantly higher SM22 staining intensity when in contact with hiPSC-ECs (Figures 2F and 2I), which indicated that heterotypic cell-cell contact in VoC culture could further promote mural cell maturation. Furthermore, long-term hiPSC-VoC culture resulted in an increase over time in the percentage of mural cells located close to the hiPSC-EC vessel wall (Figures S2E and S2F).

Assessment of hiPSC-VSMC Ca^{2+} dynamics in the VoC model

To further assess the functionality of hiPSC-VSMCs in the hiPSC-VoC, we measured intracellular Ca^{2+} release in hiPSC-VSMCs engineered to express an ultra-sensitive Ca^{2+} sensor (GCaMP6f) (Chen et al., 2013). First, hiPSC-derived neural crest (NC) intermediates were transduced with a lentiviral vector (LV) expressing GCaMP6f (Figure S3A). Transduction of hiPSC-NC cells with LVs encoding either enhanced green fluorescent protein or GCaMP6f did not change expression of the surface marker CD271 (Figure S3B). hiPSC-VSMCs engineered to express GCaMP6f showed no intracellular Ca^{2+} release upon perfusion with medium only (pre-stimulated, Figures S3C and S3D) and similar intracellular Ca^{2+} release upon stimulation with the vasoconstrictor endothelin-I (ET-I) (post-stimulated, Figures S3C and S3D) to Fluo-4-labeled

Figure 2. Quantitative assessment of the structural proprieties of hiPSC-VSMCs and primary mural cells in VoC

(A) Representative immunofluorescence images of microvascular network showing the hiPSC-ECs (magenta; agglutinin) and mural cells (green; SM22) derived vasculature unit spanning the complete length of microfluidic channel. Images showing hiPSC-ECs cultured with hiPSC-VSMCs, HBVSMCs, or HBVPs, respectively (10×). Scale bars, 200 μm.

(B) Representative confocal images of microvascular network showing hiPSC-ECs (gray; agglutinin) and mural cells (green; SM22). Images displaying xyz (i), xy (ii), and yz cross-sectional perspectives (iii). Images showing hiPSC-ECs cultured with hiPSC-VSMCs, HBVSMCs, or HBVPs, respectively (40×). Scale bars, 100 μm.

(C–E) Quantification of the percentage of mural cells associated with the hiPSC-EC lumen (% mural cells localized at the vascular network) (C), mean mural cell length (μm) (D), and mural cell circularity factor (the circle is 1) (E) in hiPSC-VSMCs, HBVPs, and HBVSMCs. Data are shown as ±SD from N = 3, n = 6; three independent experiments with two microfluidic channels per experiment.

(F) (Top) Representative confocal images of microvascular network showing hiPSC-ECs (gray; mCherry) and mural cells (red; SM22). (Bottom) Representative surface-rendered objects of confocal images showing microvascular network (gray; mCherry) and mural cells (colour-coded scale representing SM22 intensity). Images showing hiPSC-ECs cultured with hiPSC-VSMCs, HBVSMCs, or HBVPs, respectively (40×). Scale bars, 100 μm.

(G–I) Quantification of normalized mean cell SM22 intensity (G), number of SM22 + cells (H), and normalized mean cell SM22 intensity of mural cells in contact with hiPSC-ECs (I) in hiPSC-VSMCs, HBVPs and HBVSMCs. Intensity was normalized to hiPSC-VSMCs. Data are shown as ±SD from N = 3, n = 6; three independent experiments with two microfluidic channels per experiment.

One-way ANOVA (C–E and G–H) and two-way ANOVA (I) with Tukey's multiple comparison. *$p < 0.05$, **$p < 0.001$, ***$p < 0.0001$.

See also Figures S2E and S2F.

hiPSC-VSMCs (Halaidych et al., 2019). Next, intracellular Ca^{2+} release in hiPSC-VSMCs in the microvascular network was examined prior to- (basal state) and after medium refreshment on day 7. Significantly higher GCaMP6f fluorescence was observed across the entire microvascular network after medium refreshment (Figures 3A and 3B; Video S2). We then combined medium refreshment with ET-1 stimulation (1 μM, Figure 3C; Video S2). To compare the divergence in Ca^{2+} responses, the average fluorescence intensity of regions of interest over time (F/F_0) was examined by adapting previous methods (Halaidych et al., 2019) (Figure 3D). Comparison of Ca^{2+} kinetic parameters, measured at the half-maximum level $(F/F0)_{max}$, showed significantly higher amplitudes (F/F_0, Figure 3E), intensity over time (AUC [F*s/F0], Figure 3F) and duration, and slower decay of the Ca^{2+} transient without changes in the time to peak (s, Figures 3G–3I) upon ET-I stimulation.

Modeling hiPSC-derived EC-VSMC crosstalk in the VoC model

To demonstrate the potential utility of hiPSC-VoC for disease modeling, we examined the loss of EC-VSMC crosstalk upon blocking NOTCH signaling using the small-molecule γ-secretase inhibitor DAPT (10 μM). The addition of DAPT (10 μM, day 5–7) affected overall microvascular network architecture (Figure S4A) with significant changes in the mean vessel diameter although vessel density was similar between the groups (Figures S4B and S4C). DAPT supplementation had no significant effect on the percentage of hiPSC-VSMCs associated with the hiPSC-EC lumen (% mural cells localized at the vascular network, Figures 4A and 4B). However, hiPSC-VSMCs showed a significant decrease in the cell length (μm, Figure 4C) with a significant increase in cell circularity (Figure 4D). Analysis of the contractile marker SM22 in hiPSC-VSMCs showed a significantly lower normalized mean SM22 intensity (Figures 4E and 4F) while no change in the total number of SM22 + cells was observed, following addition of DAPT to the cultures (Figure 4G).

DISCUSSION

This report describes the generation of hiPSC-derived microvascular networks composed of hiPSC-ECs and hiPSC-VSMCs. We showed that hiPSC-ECs form an interconnected microvascular network with perfusable lumens and ECM deposits as in previous microfluidic studies based on other cell sources (Belair et al., 2015; Campisi et al., 2018). We demonstrate that although hiPSC-ECs alone can form a microvascular network, inclusion of mural cells facilitates hiPSC-EC self-organization and supports vessel stability. Much like primary mural cells, hiPSC-VSMCs assumed positions surrounding the vascular wall, supporting the vessel and maintaining its functionality in 3D. Although we did not find any morphological differences in microvascular network organization and mural cell morphology between hiPSC-VSMCs and primary mural cells, expression of the contractile marker SM22 did differ between hiPSC-VSMCs and HBVSMCs. hiPSC-VSMCs and HBVPs showed lower SM22 staining intensities and higher total cell numbers in VoC culture. This could indicate that hiPSC-VSMCs are less differentiated in VoC culture. Notably, by incorporating and monitoring fluorescently tagged hiPSC-derived vascular cells (Roberts et al., 2017), we showed that the most important steps of vascular network formation and remodeling occurred in the first 7 days of VoC culture. We also showed that these microvascular networks are stable for up to 3 weeks. Vessel density increased over time, although not much remodeling occurring beyond day 7 of culture. Specifically, microvascular networks formed with primary HBVSMCs showed the highest increase in vessel density, indicating that an increase in hiPSC-VSMC number observed on day 7 might be advantageous for supporting long-term VoC culture. Moreover, we demonstrated that hiPSC-VSMCs in the VoC were responsive to vasoactive stimulation by quantifying changes in Ca^{2+} kinetic parameters. We noted that hiPSC-VSMCs were activated simply after medium refreshment, an important consideration for proper experimental control. It seemed likely this was mediated by factors in the fresh media since shear-stress during gravity-mediated flow in the VoC was too slow to be stimulatory. In addition, we confirmed more pronounced and coordinated hiPSC-VSMC intracellular Ca^{2+} release following addition of the vasoconstrictor ET-I.

Conventional preclinical models have shown low success in accurately predicting drug efficacy and toxicity in human trials (Van Norman, 2020). We considered it essential therefore to provide some evidence that hiPSC-VoC responded to drugs as expected and that this property was conserved across different healthy hiPSC lines and batches. This indeed was the case: under conditions of vessel maturation where the NOTCH signaling pathway coordinates heterologous cell-cell crosstalk and maintains vessel integrity, we showed that these features were disrupted in the hiPSC-VoC after inhibition by DAPT on days 5–7. In particular, DAPT addition appeared to reduce acquisition of a contractile-like identity by hiPSC-VSMCs. This notion was supported by reduced expression the contractile marker SM22 and a less elongated morphology after EC-VSMC crosstalk inhibition by DAPT. We also observed gradual reversal of vascular stability and signs of vessel regression, reflecting what is known about dysfunctional EC-VSMC crosstalk in developed vessels *in vivo* (Kerr et al., 2016).

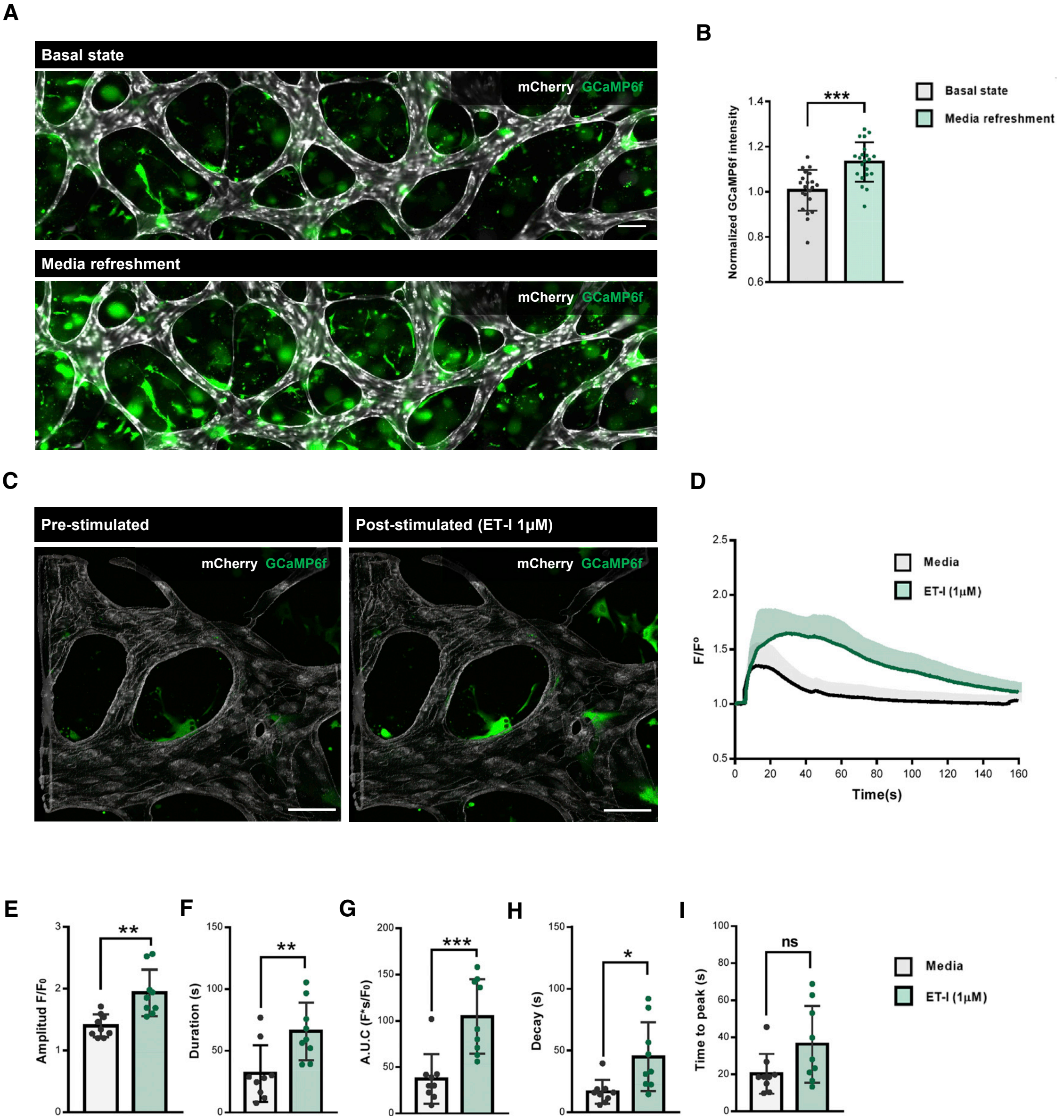

Figure 3. Analysis of hiPSC-VSMCs Ca^{2+} dynamics in VoC

(A) Representative immunofluorescent images of intracellular Ca^{2+} fluorescence showing hiPSC-ECs (gray; mCherry) and hiPSC-VSMCs (green; GCaMP6f) without- (basal state) and after EGM-2 refreshment on day 7 (10×). Scale bar, 100 μm.

(B) Normalized GCaMP6f intensity at day 7. GCaMP6f intensity was normalized to the condition prior to EGM-2 refreshment (basal state). Data are shown as ±SD of N = 3, n = 21; three independent experiments with seven microfluidic channels per experiment.

(C) Representative confocal images of intracellular Ca^{2+} fluorescence showing with hiPSC-ECs (gray; mCherry) and hiPSC-VSMCs (green; GCaMP6f) in pre- and post-stimulated (ET-I, 1 μM) states (20×). Scale bar, 100 μm.

(D) Normalized average fluorescence intensity F/F_0 in hiPSC-VSMCs expressing GCaMP6f. Medium channels were gravity-flow perfused with EGM-2 alone or containing ET-I (1 μM). Stimulation time point is set as t = 5(s). Data are shown as ±SD of N = 3, n = 9; three independent experiments with three microfluidic channels per experiment.

(E–I) Ca^{2+} transient parameters: amplitude F/F_0 (E), duration (s) (F), area under the curve (AUC, $F*s/F_0$) (G), time to peak (s) (H), and decay (s) (I) of channels gravity-flow perfused with EGM-2 alone or containing ET-I (1 μM). Data are shown as ±SD of N = 3, n = 9; three independent experiments with three microfluidic channels per experiment.

Paired (B) Student's t test. Wilcoxon-Mann-Whitney test (E–I). $*p < 0.05$, $**p < 0.001$, $***p < 0.0001$; ns, not significant.

See also Figure S3 and Video S2.

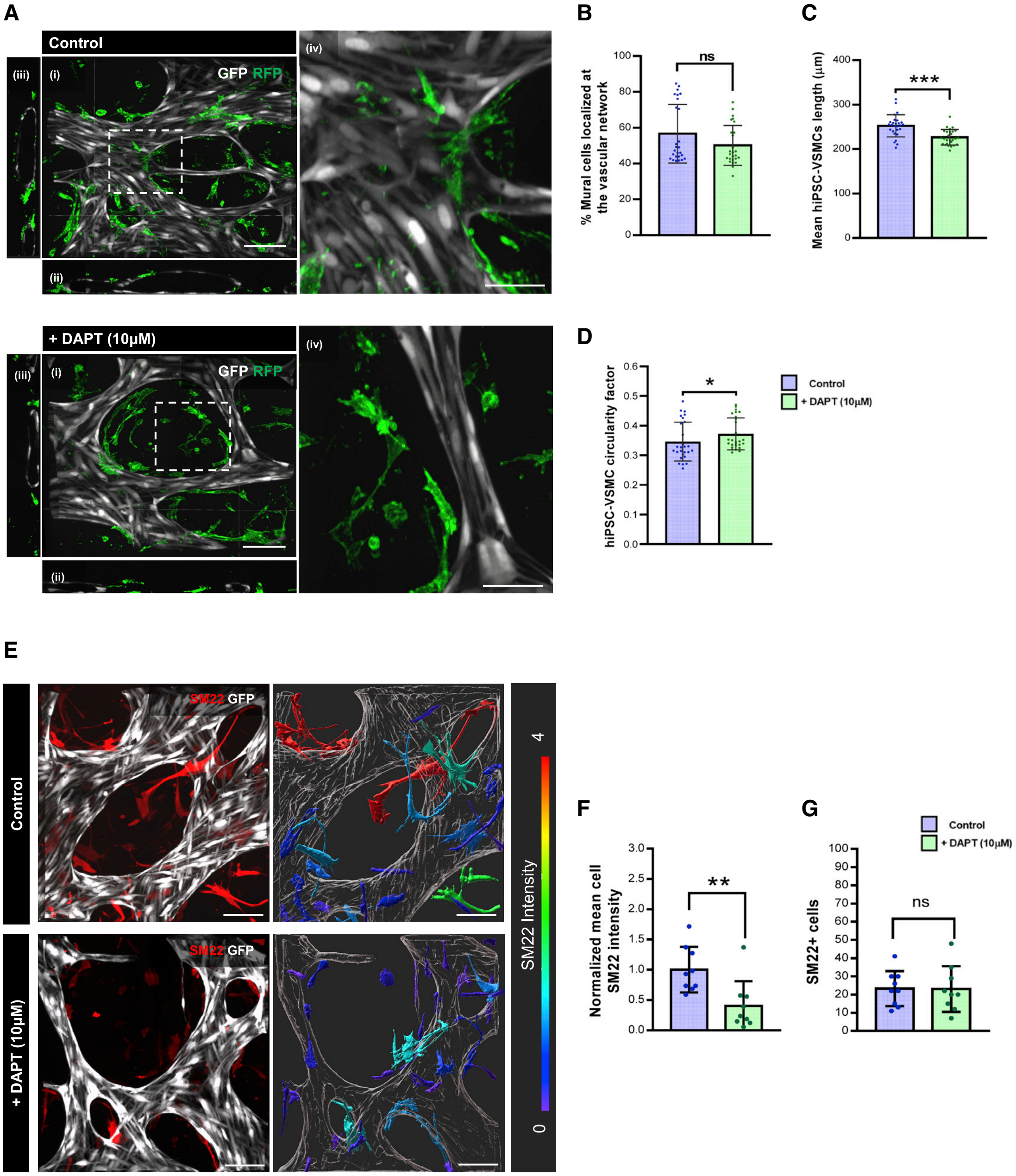

Figure 4. Modeling loss of EC-VSMC crosstalk in VoC

(A) Representative confocal images of microvascular network showing in hiPSC-ECs (gray; GFP) and hiPSC-VSMCs (green; RFP) in control and DAPT (10 μM) supplemented conditions. Images displaying xyz (i), xy, (ii), yz cross-sectional perspectives (iii), and enlargements of white framed areas (iv) (40×). Scale bars, 100 μm in (i–iii) and 50 μm in (iv).

(B–D) Quantification of the percentage of hiPSC-VSMCs associated with the hiPSC-EC lumen (% mural cells localized at the vascular network) (B), mean hiPSC-VSMCs length (μm) (C), and hiPSC-VSMC circularity factor (the circle is 1) (D) in control and DAPT (10 μM) supplemented conditions at day 7. Data are shown as ±SD from N = 3, n = 27; three independent experiments with nine microfluidic channels per experiment.

(E) (Left) Representative confocal images of microvascular network showing hiPSC-ECs (gray; mCherry) and hiPSC-VSMCs cells (red; SM22). (Right) Representative surface-rendered objects of confocal images showing microvascular network (gray; mCherry) and hiPSC-VSMCs (colour-coded scale representing SM22 intensity) in control and DAPT (10 μM) supplemented conditions (40×). Scale bars, 100 μm.

(legend continued on next page)

In summary, we have demonstrated several advantages of this hiPSC-VoC model above existing models: (1) the possibility of studying genetic vascular diseases with patient-specific lines; (2) evaluation of real-time changes in vascular structure and function by incorporating fluorescently tagged cells; (3) the ability to measure and quantify effects of drugs affecting EC-mural cell crosstalk with view to modulating vessel stability and integrity. Nevertheless, the model could be further improved by: (1) introducing physiological (rather than gravity-driven) flow which would recapitulate vessel shear forces and geometry in healthy and disease environments and (2) the addition of other hiPSC-derived cells such as monocytes or astrocytes which would allow mimicking of (isogenic) inflammatory reactions or features of the brain.

In conclusion, the hiPSC-VoC model described in this paper will be useful to study and quantify changes in the vascular architecture and function during vascular development or upon drug treatment. Clinically, this may translate into a better understanding of vascular disease conditions and predicting drug efficiency.

EXPERIMENTAL PROCEDURES

Full details are provided in supplemental experimental procedures.

hiPSC lines

Research on hiPSC was approved by the medical ethical committee at Leiden University Medical Center, the Netherlands. A detailed list of the hiPSC lines and batches used for each experiment is provided in Table S2.

Differentiation of hiPSC-ECs and hiPSC-VSMCs

hiPSC differentiation to ECs was performed as described previously (Orlova et al., 2014a, 2014b). hiPSC differentiation to VSMC was performed as previously described (Halaidych et al., 2019).

Setting up VoCs

hiPSC-ECs and mural cells were prepared prior to incorporation in VoCs as described in supplemental experimental procedures. Commercially available microfluidic chips with one gel channel and two media channels (AIM Biotech) were used. Cells were resuspended and combined to obtain 10×10^6 hiPSC-ECs/mL and 2×10^6 mural cells/mL (5:1 ratio). Three different mural cell suspensions were tested in combination with hiPSC-ECs: (1) hiPSC-VSMCs, (2) HBVSMCs, and (3) HBVPs. Cell were resuspended in EGM-2 supplemented with Thrombin (4 U/mL) and then gently mixed with fibrinogen (final concentration 3 mg/mL, Sigma) at 1:1 vol ratio. Cell/hydrogel mixture was quickly loaded into the middle gel-loading channel of the microfluidic chip. Chips were incubated at room temperature for 15 min before the addition of EGM-2 supplemented with VEGF (50 ng/mL) to both flanking media channels. The γ-secretase inhibitor DAPT (10 μM) was also added to the medium on day 1 for 24 h. Gravity-driven flow was induced by the addition of 100 μL medium to the right media ports and 50 μL media to left media ports. Medium was refreshed daily.

Statistical analysis

Statistical analyses were performed using GraphPad Prism 9 software. Normality of the data was evaluated by the D'Agostino-Pearson test. One-way and two-way ANOVA with Tukey's multiple comparison test was used for the analysis of three groups. For paired or unpaired analysis of two groups, either Student's t test or Wilcoxon-Mann-Whitney test was used. Analyses are indicated in the figure legends. The data are reported as mean ± SD. Statistical significance was defined as $p < 0.05$.

SUPPLEMENTAL INFORMATION

Supplemental information can be found online at https://doi.org/10.1016/j.stemcr.2021.08.003.

AUTHOR CONTRIBUTIONS

Conceptualization, V.V.O.; methodology, M.V.C., A.C., and V.V.O.; software, A.C.; validation, A.C. and M.V.C.; formal analysis, A.C. and M.V.C.; investigation, A.C., M.V.C., and F.E.v.d.H.; visualisation, M.V.C. and A.C.; resources, A.A.F.d.V. and V.V.O.; writing – original draft, M.V.C., A.C., C.L.M., and V.V.O.; writing – review & editing, M.V.C., A.C., S.A.J.L.O., C.L.M., and V.V.O.; supervision, S.A.J.L.O, C.L.M., and V.V.O.; project administration, V.V.O.; funding acquisition, A.C., S.A.J.L.O., C.L.M., and V.V.O.

CONFLICT OF INTERESTS

The authors declare no competing interests.

ACKNOWLEDGMENTS

The LUMC human iPSC Hotel for generation and characterization of hiPSC lines; LUMC's confocal imaging facility; Cindy Bart, Sven Dekker and Juan Zhang for LV production; Oleh Halaidych for useful discussion on hiPSC-VSMCs. The Allen Cell Collection, available from Coriell Institute for Medical Research, provided materials. Images were generated using Biorender.com.

This work was supported by the Netherlands Organisation for Health Research and Development (ZonMw): PTO 446002501 and VIDI 91717325; the Netherlands Organ-on-Chip Initiative which is an NWO Gravitation project (024.003.001) funded by the Ministry of Education, Culture and Science of the government of the Netherlands; European Research Council (ERCAdG 323182 STEMCARDIOVASC); the European Union's Horizon

(F and G) Quantification of normalized mean cell SM22 intensity (F) and number of SM22 + cells (G). Intensity was normalized to control condition. Data are shown as ±SD from N = 3, n = 9; three independent experiments with three microfluidic channels per experiment. Wilcoxon-Mann-Whitney test. *$p < 0.05$, **$p < 0.001$, ***$p < 0.0001$; ns, not significant.
See also Figure S4.

2020 research and innovation program under the Marie Sklodowska Curie grant agreement no. 707404.

Received: March 1, 2021
Revised: August 5, 2021
Accepted: August 6, 2021
Published: September 2, 2021

REFERENCES

Belair, D.G., Whisler, J.A., Valdez, J., Velazquez, J., Molenda, J.A., Vickerman, V., Lewis, R., Daigh, C., Hansen, T.D., Mann, D.A., et al. (2015). Human vascular tissue models formed from human induced pluripotent stem cell derived endothelial cells. Stem Cell Rev. Rep. *11*, 511–525.

Berry, C., Sidik, N., Pereira, A.C., Ford, T.J., Touyz, R.M., Kaski, J.C., and Hainsworth, A.H. (2019). Small-vessel disease in the heart and brain: current knowledge, unmet therapeutic need, and future directions. J. Am. Heart Assoc. *8*, e011104.

Campisi, M., Shin, Y., Osaki, T., Hajal, C., Chiono, V., and Kamm, R.D. (2018). 3D self-organized microvascular model of the human blood-brain barrier with endothelial cells, pericytes and astrocytes. Biomaterials *180*, 117–129.

Chen, T.W., Wardill, T.J., Sun, Y., Pulver, S.R., Renninger, S.L., Baohan, A., Schreiter, E.R., Kerr, R.A., Orger, M.B., Jayaraman, V., et al. (2013). Ultrasensitive fluorescent proteins for imaging neuronal activity. Nature *499*, 295–300.

Cochrane, A., Albers, H.J., Passier, R., Mummery, C.L., van den Berg, A., Orlova, V.V., and van der Meer, A.D. (2019). Advanced in vitro models of vascular biology: human induced pluripotent stem cells and organ-on-chip technology. Adv. Drug Deliv. Rev. *140*, 68–77.

Duval, K., Grover, H., Han, L.H., Mou, Y., Pegoraro, A.F., Fredberg, J., and Chen, Z. (2017). Modeling physiological events in 2D vs. 3D cell culture. Physiology (Bethesda) *32*, 266–277.

Halaidych, O.V., Cochrane, A., van den Hil, F.E., Mummery, C.L., and Orlova, V.V. (2019). Quantitative analysis of intracellular Ca(2+) release and contraction in hiPSC-derived vascular smooth muscle cells. Stem Cell Reports *12*, 647–656.

Halaidych, O.V., Freund, C., van den Hil, F., Salvatori, D.C.F., Riminucci, M., Mummery, C.L., and Orlova, V.V. (2018). Inflammatory responses and barrier function of endothelial cells derived from human induced pluripotent stem cells. Stem Cell Reports *10*, 1642–1656.

Kerr, B.A., West, X.Z., Kim, Y.W., Zhao, Y., Tischenko, M., Cull, R.M., Phares, T.W., Peng, X.D., Bernier-Latmani, J., Petrova, T.V., et al. (2016). Stability and function of adult vasculature is sustained by Akt/Jagged1 signalling axis in endothelium. Nat. Commun. *7*, 10960.

Orlova, V.V., Drabsch, Y., Freund, C., Petrus-Reurer, S., van den Hil, F.E., Muenthaisong, S., Dijke, P.T., and Mummery, C.L. (2014a). Functionality of endothelial cells and pericytes from human pluripotent stem cells demonstrated in cultured vascular plexus and zebrafish xenografts. Arterioscler. Thromb. Vasc. Biol. *34*, 177–186.

Orlova, V.V., van den Hil, F.E., Petrus-Reurer, S., Drabsch, Y., Ten Dijke, P., and Mummery, C.L. (2014b). Generation, expansion and functional analysis of endothelial cells and pericytes derived from human pluripotent stem cells. Nat. Protoc. *9*, 1514–1531.

Owens, G.K., Kumar, M.S., and Wamhoff, B.R. (2004). Molecular regulation of vascular smooth muscle cell differentiation in development and disease. Physiol. Rev. *84*, 767–801.

Roberts, B., Haupt, A., Tucker, A., Grancharova, T., Arakaki, J., Fuqua, M.A., Nelson, A., Hookway, C., Ludmann, S.A., Mueller, I.A., et al. (2017). Systematic gene tagging using CRISPR/Cas9 in human stem cells to illuminate cell organization. Mol. Biol. Cell *28*, 2854–2874.

Samuel, R., Duda, D.G., Fukumura, D., and Jain, R.K. (2015). Vascular diseases await translation of blood vessels engineered from stem cells. Sci. Transl. Med. *7*, 309rv306.

Tronolone, J.J., and Jain, A. (2021). Engineering new microvascular networks on-chip: ingredients, assembly, and best practices. Adv. Funct. Mater. *31*, 2007199.

van Dijk, C.G.M., Brandt, M.M., Poulis, N., Anten, J., van der Moolen, M., Kramer, L., Homburg, E., Louzao-Martinez, L., Pei, J., Krebber, M.M., et al. (2020). A new microfluidic model that allows monitoring of complex vascular structures and cell interactions in a 3D biological matrix. Lab Chip *20*, 1827–1844.

van Duinen, V., Zhu, D., Ramakers, C., van Zonneveld, A.J., Vulto, P., and Hankemeier, T. (2019). Perfused 3D angiogenic sprouting in a high-throughput in vitro platform. Angiogenesis *22*, 157–165.

Van Norman, G.A. (2020). Limitations of Animal studies for predicting toxicity in clinical trials: Part 2: potential alternatives to the use of animals in preclinical trials. JACC Basic Transl. Sci. *5*, 387–397.

Stem Cell Reports, Volume *16*

Supplemental Information

Engineered 3D vessel-on-chip using hiPSC-derived endothelial- and vascular smooth muscle cells

Marc Vila Cuenca, Amy Cochrane, Francijna E. van den Hil, Antoine A.F. de Vries, Saskia A.J. Lesnik Oberstein, Christine L. Mummery, and Valeria V. Orlova

Inventory of Supplemental Information

Supplemental Figures and Legends:

Figure S1. Self-organization of VoC vascular network with hiPSC-ECs alone. Related to Figure 1.
Figure S2. Self-organization of VoC vascular network with prolonged culture. Related to Figure 1 & 2.
Figure S3. Characterization of hiPSC-VSMCs expressing GCaMP6f LV. Related to Figure 3.
Figure S4. Characterization of microvascular network upon EC-VSMC cross-talk modelling in VoC. Related to Figure 4.

Supplemental Tables:

Table S1. Flow experimental details.
Table S2. Complete list of hiPSC lines and batches used per experiment.

Supplemental Experimental Procedures

Supplemental References

Supplemental Videos:

Video S1. Perfusion of fluorescent beads (top panels) and FITC-Dextran (70 kDa)(bottom panels). Related to Figure 1.
Video S2. Intracellular Ca^{2+} Release in hiPSC-VSMC in VoC upon medium refreshment (left panel) and upon ET-I stimulation (right panel). Related to Figure 3.

Supplemental Figures and Legends

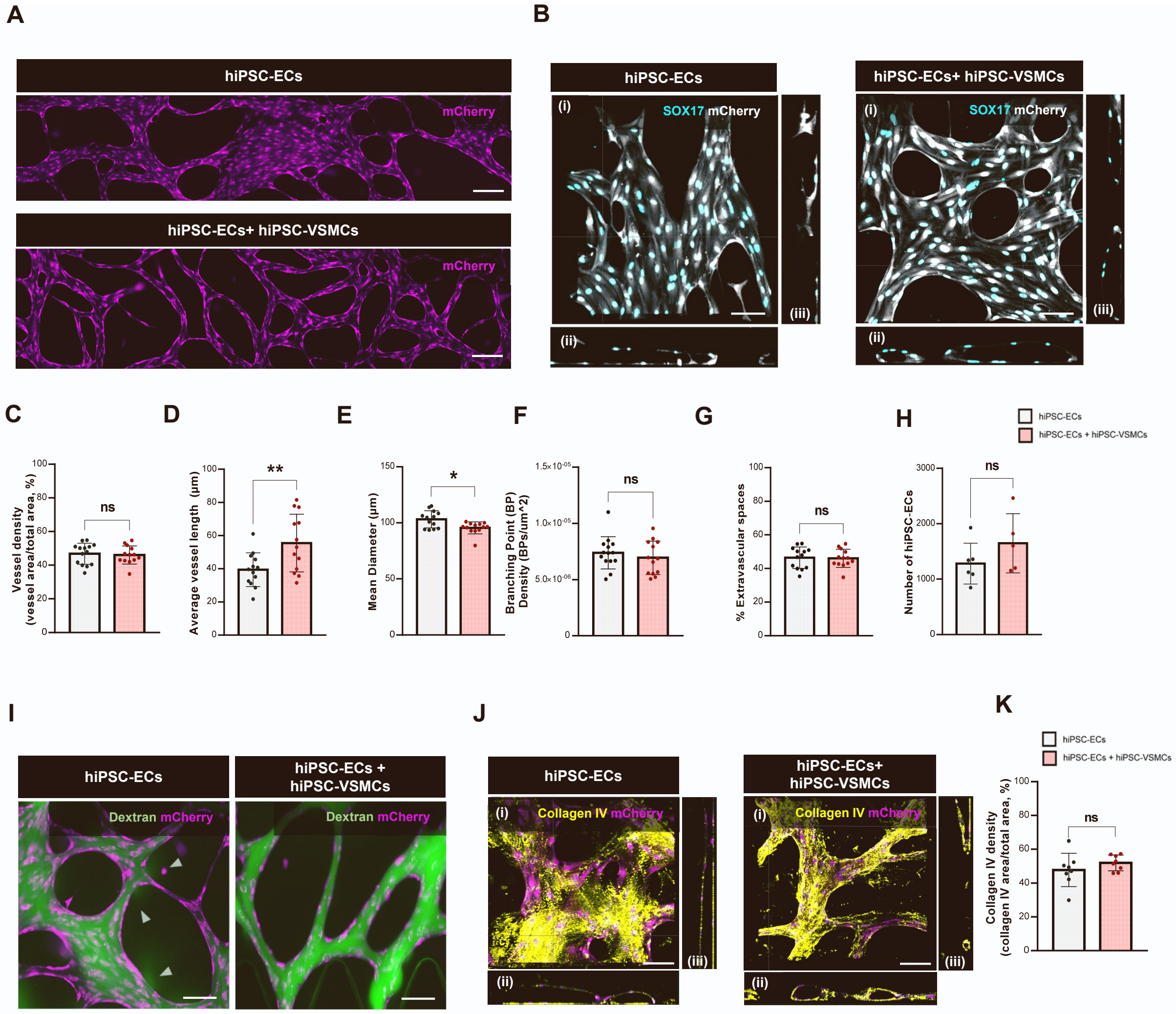

Figure S1. Self-organization of VoC vascular network with hiPSC-ECs alone. Related to Figure 1. (A) Representative immunofluorescence images of microvascular network showing hiPSC-ECs (magenta; mCherry). Images showing hiPSC-ECs cultured alone or with hiPSC-VSMCs respectively. 10x, scale bars 200 μm. (B) Representative confocal images of microvascular network showing hiPSC-ECs (grey; mCherry) and hiPSC-EC nuclei (cyan; SOX17). Images displaying xyz (i), xy (ii) and yz cross-sectional perspectives (iii). Images showing hiPSC-ECs cultured alone or with hiPSC-VSMCs respectively. 40x, scale bars 100 μm. (C-G) Quantification of vessel density (%, C), average vessel length (μm, D), mean diameter (μm, E), branching point (BP) density (BPs/μm2, F) and extravascular spaces (%, G) from hiPSC-ECs cultured alone or with hiPSC-VSMCs respectively are shown. Data are shown as ±SD from N=3, n=13; three independent experiments with three to six microfluidic channels per experiment. (H) Quantification of number of hiPSC-ECs from hiPSC-ECs cultured alone or with hiPSC-VSMCs respectively are shown. Data are shown as ±SD from N=3, n=6; three independent experiments with duplicates microfluidic channels per experiment. (I) Representative Immunofluorescence images showing hiPSC-ECs (magenta; mCherry) and perfusion of 70 kDa FITC-Dextran (green). Images showing hiPSC-ECs cultured alone or with hiPSC-VSMCs respectively. 10x, scale bars 100 μm. (J) Representative confocal images of microvascular network showing hiPSC-ECs (magenta; mCherry) and ECM (yellow; Collagen IV). Images displaying xyz (i), xy (ii) and yz cross-sectional perspectives (iii). Images showing hiPSC-ECs cultured alone or with hiPSC-VSMCs respectively. 40x, scale bars 100 μm. (K) Quantification of Collagen IV density (%) from hiPSC-ECs cultured alone or with hiPSC-VSMCs respectively are shown. Data are shown as ±SD from N=3, n=8; three independent experiments with two to three microfluidic channels per experiment. Wilcoxon-Mann-Whitney test. *p < 0.05, ** p < 0.001, ns = not significant.

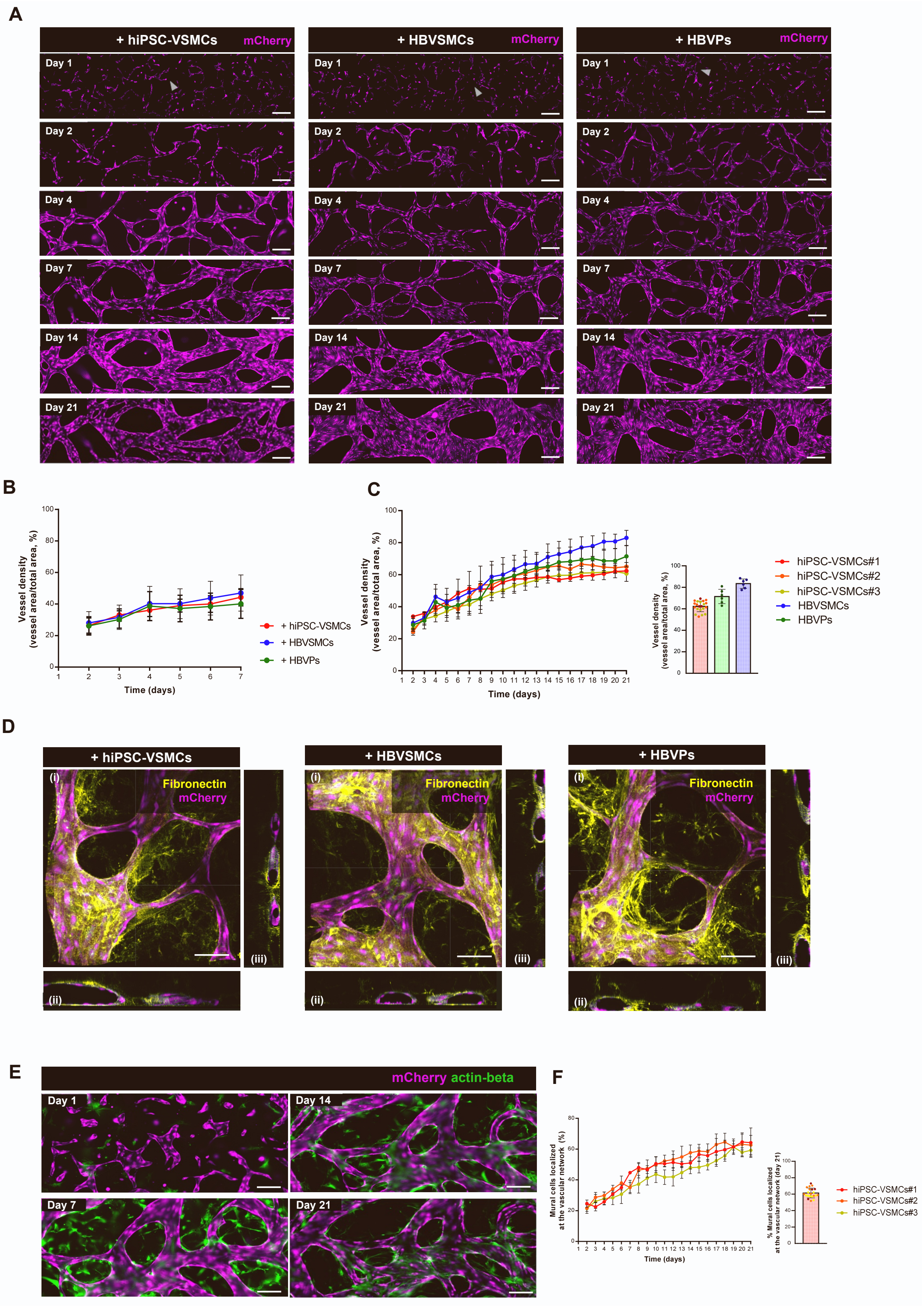

A
+ hiPSC-VSMCs mCherry
+ HBVSMCs mCherry
+ HBVPs mCherry
Day 1
Day 2
Day 4
Day 7
Day 14
Day 21
B
Vessel density (vessel area/total area, %)
Time (days)
+ hiPSC-VSMCs
+ HBVSMCs
+ HBVPs
C
Vessel density (vessel area/total area, %)
Time (days)
hiPSC-VSMCs#1
hiPSC-VSMCs#2
hiPSC-VSMCs#3
HBVSMCs
HBVPs
D
+ hiPSC-VSMCs
+ HBVSMCs
+ HBVPs
Fibronectin
mCherry
(i)
(ii)
(iii)
E
mCherry actin-beta
Day 1
Day 14
Day 7
Day 21
F
Mural cells localized at the vascular network (%)
Time (days)
% Mural cells localized at the vascular network (day 21)
hiPSC-VSMCs#1
hiPSC-VSMCs#2
hiPSC-VSMCs#3

Figure S2. Self-organization of VoC vascular network with prolonged culture. Related to Figure 1 & 2. (A) Daily immunofluorescence images (day 1, 2, 4, 7, 14, 21) showing hiPSC-ECs (magenta; mCherry) vascular cell self-organization, vasculogenesis and vascular remodelling over time. Images showing hiPSC-ECs cultured with hiPSC-VSMCs, HBVSMCs or HBVPs respectively. White arrowheads show vacuole formation at day 1. 10x, scale bars 200 µm. (B) Quantification of daily timepoints of the percentage of vessel density from day 2 (after formation of interconnected network) until day 7 with hiPSC-ECs cultured with hiPSC-VSMCs, HBVSMCs or HBVPs respectively. Data are shown as ±SD from N=3, n=20; three independent experiments with six to eight microfluidic channels per experiment. (C) Quantification of daily timepoints of the percentage of vessel density from day 2 (after formation of interconnected network) until day 21 with hiPSC-ECs cultured with hiPSC-VSMCs, HBVSMCs or HBVPs respectively. Quantification of vessel density at day 21 is shown as scatterplot with bar. Data are shown as ±SD from N=3, n=21; three independent experiments with three to nine microfluidic channels per experiment (hiPSC-VSMCs; AICS-0016 [#1-2] and LUMC0054iCTRL [#3]). N=1, n=6; one independent experiment with six microfluidic channels per experiment (HBVSMCs and HBVPs). (D) Representative confocal images of microvascular network showing hiPSC-ECs (magenta; mCherry) and ECM (yellow; Fibronectin). Images displaying xyz (i), xy (ii) and yz cross-sectional perspectives (iii). Images showing hiPSC-ECs cultured with hiPSC-VSMCs, HBVSMCs or HBVPs respectively. 40x, scale bars 100 µm. (E) Daily immunofluorescence images (day 1, 7, 14, 21) showing vascular cell self-organization and vascular remodelling over time. hiPSC-ECs (magenta; mCherry) and hiPSC-VSMCs (green; actin-beta). 20x, scale bar 200 µm. (F) Quantification of the percentage of mural cells associated with the hiPSC-EC lumen (% mural cells localized at the vascular network) from day 2 (after formation of interconnected network) until day 21. Quantification of the percentage of mural cells associated with the hiPSC-EC lumen at day 21 is shown as scatterplot with bar. Data are shown as ±SD from N=3, n=21; three independent experiments with three to nine microfluidic channels per experiment (hiPSC-VSMCs; AICS-0016 [#1-2] and LUMC0054iCTRL [#3]).

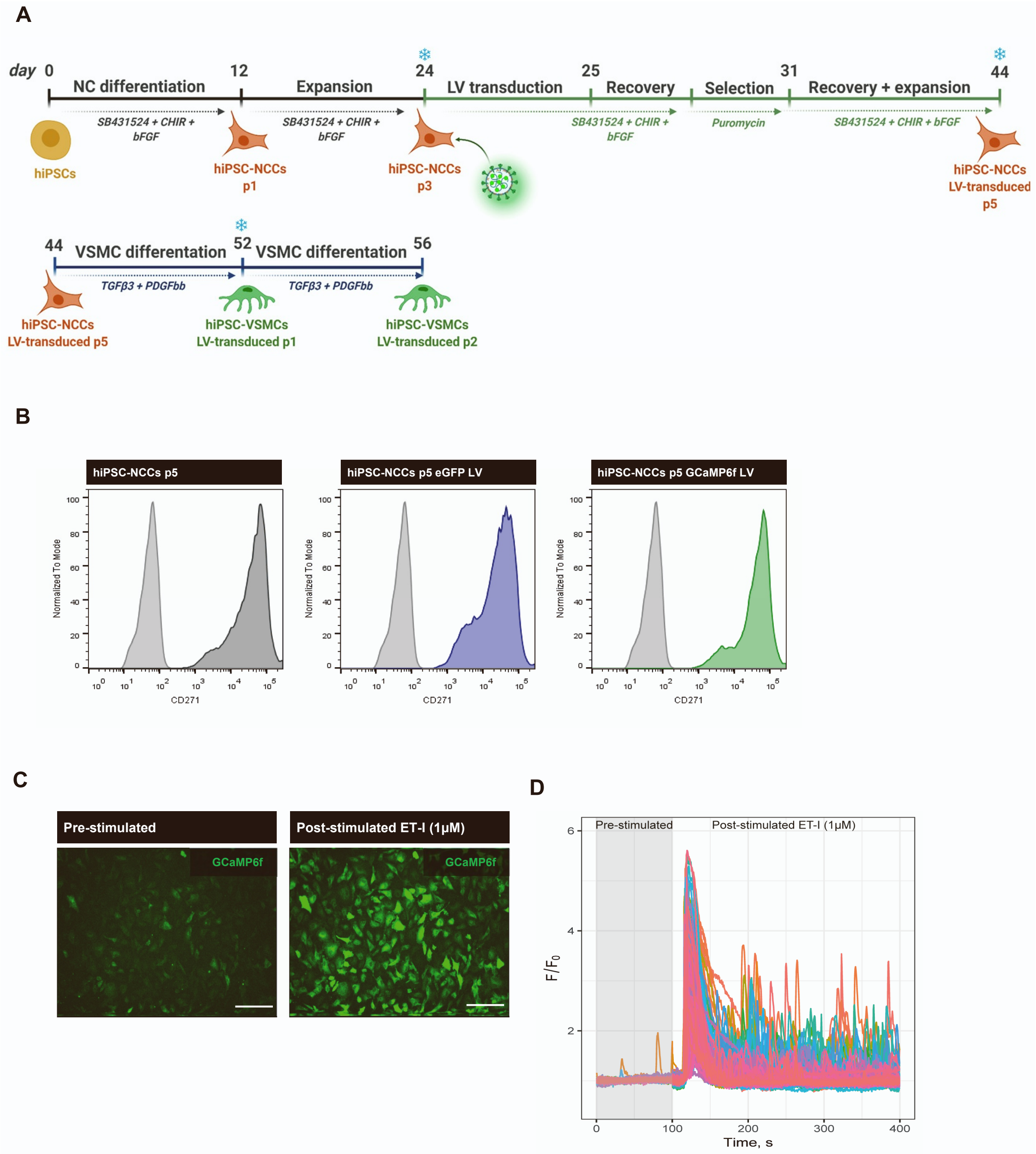

Figure S3. Characterization of hiPSC-VSMCs expressing GCaMP6f LV. Related to Figure 3. (A) Schematic illustration of the LV-transduction hiPSC-NC cells (hiPSC-NCCs) and hiPSC-VSMCs differentiation protocol. (B) FACS analysis of surface expression of CD271 in non-transduced hiPSC-NCCs (black filled histograms), hiPSC-NCCs expressing control LV (eGFP LV; blue filled histograms) or hiPSC-NCCs expressing GCaMP6f LV (GCaMP6f LV; green filled histograms). (C) Representative image from time-lapse of intracellular Ca^{2+} fluorescence in hiPSC-VSMCs (green; GCaMP6f) from in pre- and post- stimulated (ET-I 1 μM) states respectively. 10x, scale bar 200 μm. (D) Normalized average fluorescence intensity (F/F_0) within distinct region of interests (ROIs) over the time of the image sequence measured in hiPSC-VSMCs expressing GCaMP6f LV (one representative experiment). Each individual trace corresponds to one detected and tracked ROI. Time window marked in grey represents medium perfusion period (pre-stimulated state, 0–100 s). Post-stimulated state indicates a time of perfusion with ET-I (1 μM, 100-400 s).

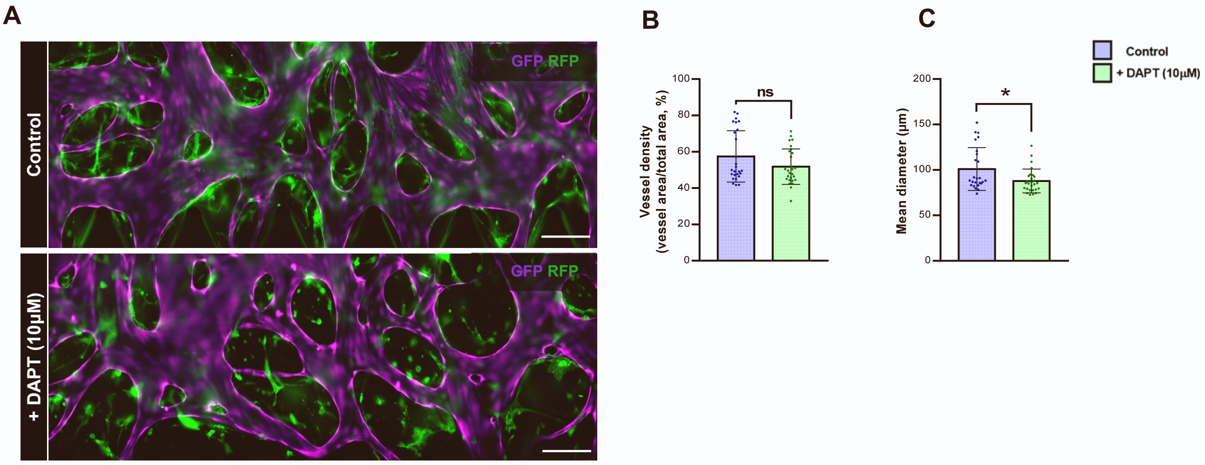

Figure S4. Characterization of microvascular network upon EC-VSMC cross-talk modelling in VoC. Related to Figure 4. (A) Representative immunofluorescence images of microvascular network showing hiPSC-ECs (magenta; GFP) and hiPSC-VSMCs (green; RFP) in control and DAPT (10 µM) supplemented conditions. 20x, scale bars 200 µm. (B-C) Quantification of the vessel density (%, B) and diameter (µm, C) of microfluidic channels in control and DAPT (10 µM) supplemented conditions at day 7. Data are shown as ±SD from N=3, n=27; three independent experiments with nine microfluidic channels per experiment. *p < 0.05, ns = not significant.

Supplemental Tables

Table S1. Flow experimental details.

Parameters	Values
Vessel length (μm)	597 – 934
Vessel diameter (μm)	75
Time for bead displacement (s)	4,5 – 20
Fluid proprieties (g/cm^3)	1
Wall shear stress (dyne/cm^2)	0,056 - 0,14
Flow velocity (mm/s)	0,05 – 0,13
Absolute Pressure difference (mBar)	0,5
Time flow convection stops (min)	14-18
Time flow is re-stablished (h)	24

Table S2. Complete list of hiPSC lines and batches used per experiment.

**Figure**	**hiPSC-ECs**		**hiPSC-VSMCs**	
	Line	Number of Batches	Line	Number of Batches
1F-K; 2C-E	LUMC0020iCTRL	1	LUMC0020iCTRL	1
	LUMC0054iCTRL	2	LUMC0054iCTRL	2
1M; 2G-I	LUMC0054iCTRL	2	LUMC0054iCTRL	3
	NCRM-1	1		
S1C-H, K	NCRM-1	3	AICS-0016	1
			LUMC0054iCTRL	2
S2B-C, F	NCRM-1	3 (+hiPSC-VSMCs);<br>1 (+ HBVPs/HBVSMCs)	LUMC0054iCTRL	1
			AICS-0016	2
3B, D-I	NCRM-1	3	LUMC0054iCTRL	3
4B-D, F-G; S4B-C	NCRM-1	3	AICS-0016	1
			AICS-0054	1
			LUMC0054iCTRL	1

Supplemental Experimental Procedures

hiPSC lines and maintenance

hiPSCs were maintained on recombinant vitronectin-coated plates in TeSR-E8, all from STEMCELL Technologies, according to the manufacturer's instructions. The following hiPSC lines were used: LUMC0054iCTRL (generated from kidney epithelial cells isolated from urine, http://hpscreg.eu/cell-line/LUMCi001-A) (Halaidych et al., 2018) and LUMC0020iCTRL (generated from skin fibroblasts, https://hpscreg.eu/cell-line/LUMCi028-A) (Zhang et al., 2014). NIH Center for Regenerative Medicine hiPSC line (NCRM-1, generated from CD34+ cord blood cells, https://hpscreg.eu/cell-line/CRMi003-A), obtained from RUDCR Infinite Biologics at Rutgers University, was modified in-house with a mCherry or GFP expression cassette under the human cytomegalovirus (hCMV) early enhancer/chicken β actin (CAG) promoter using a previously established protocol(Rostovskaya et al., 2012). The Allen Cell Collection lines: AICS-0016 (generated from skin fibroblasts, https://hpscreg.eu/cell-line/UCSFi001-A-3) with mEGFP insertion site at ACTB and AICS-0054 (generated from skin fibroblasts, https://hpscreg.eu/cell-line/UCSFi001-A-23) with mTagRFPT insertion site at AAVS1 were obtained from Coriell Institute for Medical Research.

Differentiation of hiPSCs towards ECs

hiPSCs were maintained in mTeSR-E8 and differentiated towards ECs using previously published protocols (Orlova et al., 2014a; Orlova et al., 2014b). For mesoderm induction (day 0-3), mTeSR-E8 medium was replaced with B(P)EL medium supplemented with 8 μM CHIR99021 (Tocris Bioscience, 4423). Cells were refreshed with vascular specification medium comprised of VEGF (50 ng/ml) and 10 µM SB431542 (Tocris Bioscience, 1614) in B(P)EL at day 3, day 6, and day 9. hiPSC-ECs were isolated on day 10 using CD31-Dynabeads™ (Thermo Fisher Scientific), as previously described (Orlova et al., 2014b; 2014a). hiPSC-ECs were expanded in complete EC growth medium comprised of Human Endothelial-serum free medium (EC-SFM) with 1% Human platelet poor serum (P2918, Sigma), VEGF (30 ng/ml) and bFGF (20 ng/ml), as described previously with minor modifications (Orlova et al., 2014a; Orlova et al., 2014b). hiPSC-ECs were expanded for additional 3-4 days post-isolation and cryopreserved using serum-free cryopreservation medium at passage number 1 (P1) (CryoStor™CS10) (StemCell Technologies, 07930).

Differentiation of hiPSCs towards VSMCs

hiPSC colonies were passaged and kept in hiPSC mTeSR-E8 and differentiated towards NCCs using previously published protocols (Halaidych et al., 2019a). After 2 days, the medium was changed to NC differentiation medium consisting of B(P)EL medium supplemented with 10 µM SB431542 (Tocris Bioscience, 1614), 1 µM CHIR99021 (Tocris Bioscience, 4423) and 10 ng/mL bFGF (Miltenyi Biotec, 130-093-842). Cells were refreshed every 2 days and kept in NC differentiation medium for 10-12 days. After 10-12 days NC cells (NCCs) were passaged with 1xTrypLE Select (Gibco, 12563029) and plated in 1:4 ratio on Matrigel-coated plates. hiPSC-NCCs were cryopreserved at passage number 3 (P3) using serum-free cryopreservation medium (CryoStor™CS10) (StemCell Technologies, 07930). NCCs were differentiated into VSMCs following a previously described protocol with minor modifications (Halaidych et al., 2019a). NCCs were plated at $3x10^4$ cells/cm^2 seeding density on 0.1% Gelatin (Sigma-Aldrich, G1890) coated plates in VSMC differentiation medium consisting of B(P)EL medium supplemented with 2 ng/mL TGF-β3 (PeproTech, 100-36E) and 10 ng/mL PDGF-BB (PeproTech, 100-14B). Cells were refreshed every 2 days and kept in VSMC differentiation medium for 8 days. Cells were passaged in a 1:4 splitting ratio at day 4. hiPSC-VSMCs were cryopreserved at passage number 1 (P1) using serum-free cryopreservation medium (CryoStor™CS10) (StemCell Technologies, 07930)

Primary mural cell culture

Human brain vascular pericytes (HBVPs) and Human brain vascular smooth muscle cells (HBVSMCs) were purchased from ScienceCell. HBVPs were cultured in Pericyte Medium (ScienceCell, 1201) supplemented with Pericyte Growth Supplement (ScienceCell, 1252) and 2% FBS. HBVSMCs were cultured in Smooth Muscle Cell Medium (ScienceCell, 1101) supplemented with Smooth Muscle Growth Supplement (ScienceCell, 1152) and 2% FBS. Cells were cryopreserved at passage number 3 (P3) using serum-free cryopreservation medium (CryoStor™CS10) (StemCell Technologies, 07930).

Cell preparation prior to VoC culture

hiPSC-ECs (P1) were thawed and cultured on gelatin-coated plates in complete EC growth medium composed of Human Endothelial-SFM (EC-SFM) with 1% platelet poor serum (PPS), VEGF (30 ng/ml) and bFGF (20 ng/ml), as described previously (Orlova et al., 2014b; 2014a) 4 days prior to VoC seeding.

hiPSC-VSMCs (P1) were thawed and cultured on gelatin-coated plates in B(P)EL medium supplemented with 2 ng/mL TGF-β3 (PeproTech, 100-36E) and 10 ng/mL PDGF-bb (PeproTech, 100-14B) 4 days prior to VoC seeding using previously described protocol with minor modifications (Protocol A, Halaidych et al., 2019). HBVPs (P3) were thawed and cultured in Pericyte Medium (ScienceCell, 1201) supplemented with Pericyte Growth Supplement (ScienceCell, 1252) and 2% FBS. HBVSMCs (P3) cultured in Smooth Muscle Cell Medium (ScienceCell, 1101) supplemented with Smooth Muscle Growth Supplement (ScienceCell, 1152) and 2% FBS. HBVPs and HBVSMCs were seeded on gelatin coated plates and cultured 4 days prior to VoC seeding.

Perfusion assessment in VoC system
For perfusion assessment in the VoC, hiPSC-ECs were labelled with 594-Agglutinin (1:600 in EGM-2, Vector laboratories) for 30 min in a CO_2 incubator to assess the vessel density after 7 days of culture. The chip was placed into the EVOS AUTO2 with an on-stage incubator for time-lapse image acquisition. First, basal fluorescence activity before the addition of fluorescent tracers was captured. Next, 70 µl of 70KDa FITC-Dextran (1:1000, Sigma) or 405-beads (1:10, Fluoro-Max Dyed Blue Aqueous Fluorescent Particles, B0200, ThermoFisher Scientific) in EGM-2 was added to one medium port and 50 µl of EGM-2 to all other media ports to induce interstitial gravity flow. Then, simultaneous image capturing at 20 fps using a 10x magnification objective was started. Confocal images were acquired to create a 3D stack using a DragonFly spinning disk (Andor) microscope with 20x magnification objective and processed using Imaris software (Bitplane, Oxford Instruments). Confocal images for dextran perfusion were acquired using a DragonFly spinning disk (Andor) microscope with 40x magnification objective and processed using Imaris 9.5 software (Bitplane, Oxford Instruments).

Flow parameters assessment in VoC system
To approximate the initial flow velocity in the microvessels, 405-beads (1:10, Fluoro-Max Dyed Blue Aqueous Fluorescent Particles, B0200, ThermoFisher Scientific) in PBS was added to the right media ports (100 µl) and to left connecting media ports (50 µl). Then, simultaneous image capturing with EVOSM7000 at 40 fps using a 4x magnification objective was started. Image stacks were imported and processed using Imaris 9.5 software (Bitplane, Oxford Instruments). 405-beads were tracked by using the 3D rendering and cell-tracking function over time. Three vascular segments were selected as ROI to obtain the track displacement length and duration of the beads. Time until convection stops was estimated when the velocity of the 405-beads in the media channel was 0. Wall shear stress, pressure and flow velocity were calculated with https://darwin-microfluidics.com/blogs/tools/microfluidic-flowrate-and-shear-stress-calculator.

Immunostaining and Microscopy
Cells in VoCs were fixed *in situ* in 4% paraformaldehyde (PFA) for 30 min at RT. Cell plasma membranes were permeabilized with 0.5% Triton X-100 for 15 min at RT and washed 3 times for 10 mins between each step with PBS, then blocking buffer (2% BSA) was added for 3 hours at RT. Primary antibodies (1:200 volume ratio in 1% BSA), against CD31 (PECAM1, Mouse M0823 DAKO), SOX17 (goat, AF1924, R&D Systems), SM22 (TAGLIN; Rabbit, ab14106, Abcam), Collagen IV (goat, AB769, Sigma), Fibronectin (Rabbit, F3648, Sigma) used to identify hiPSC-ECs, hiPSC-EC nuclei, mural cells and ECM respectively, were incubated overnight at 4 ºC. Secondary antibodies (1:300 volume ratio in 1% BSA), were incubated for 2 hours at RT after 3 times 15 min PBS washes. VoCs were imaged using EVOS M7000 using 10x magnification objective. A customised plate layout that allowed for automated imaging and stitching to produce images of complete microfluidic channel for all fluorescent channels was used. For 3D stacks, images were taken using a DragonFly spinning disk (Andor) microscope with 40x magnification objective and post-processing performed and processed using Imaris 9.5 software (Bitplane, Oxford Instruments).

Characterisation of vascular and perivascular parameters in 2D
Images from the whole microfluidic channel (acquired using EVOS) were quantified using pipelines developed on the free open source CellProfiler software (https://cellprofiler.org/) (Carpenter et al., 2006). Briefly, for EC nuclei number, pre-processing steps were applied to all images to enhance image features and a gaussian filter to reduce unspecific object identification. A Gaussian filter applied to mural cell images before object identification was used to measure object morphology and staining intensity (GCaMP6f). Two filter steps were applied to images of vascular network to reduce non-specific segmentation from cell junctions and a minimum cross-entropy thresholding method was used to produce a binarized image. For ECM area quantification, two filter steps were applied to images of Collagen IV and robust background thresholding method was used to produce a binarized image. The

binarized images from the CellProfiler output were then analyzed using the freely available ImageJ software with the plugin (https://imagej.nih.gov/ij/ ,https://imagej.net/DiameterJ) (Hotaling et al., 2015)..

Characterization of mural cell parameters in 3D
For 3D quantitative analysis, surface-rendering of individual mural cells (SM22) was performed and processed using Imaris 9.5 software (Bitplane, Oxford Instruments). Mean object SM22 intensity and number of SM22 positive objects was obtained from each 3D stack. To define the mural cells in contact with hiPSC-ECs, surface-rendering of vessel structure (CD31, mCherry and GFP) was performed and distance of SM22 objects was defined as 0 µm (contact) and >0 µm (no contact) from the object hiPSC-ECs. SM22 objects where filtered based on distance and mean object SM22 intensity was obtained.

Plasmid constructs
The lentiviral vector (LV) shuttle plasmid pLV.hCMV-IE.GCaMP6f.IRES.PurR.hHBVPRE was generated in a multistep procedure using pGP-CMV-GCaMP6f (Addgene, Watertown, MA; plasmid number 40755) and pLV.hCMV-IE.IRES.PurR.hHBVPRE as starting constructs (Neshati et al., 2014). pLV.hCMV-IE.GCaMP6f.IRES.PurR.hHBVPRE contains a human cytomegalovirus immediate-early gene (hCMV-IE) promoter driving expression of a bicistronic mRNA encoding the ultra-sensitive $[Ca^{2+}]_{cyt}$ sensor GCaMP6f (Chen et al., 2013) and *Streptomyces alboniger* puromycin-N-acetyltransferase. The LV shuttle plasmid pLV.hCMV-IE.eGFP.PurR.hHBVPRE was generated by insertion of the *Aequorea victoria* enhanced green fluorescent protein (eGFP)-encoding 754-bp SmaI×EcoRI fragment of pEGFP (Clontech - Takara Bio Europe, Saint-Germain-en-Laye, France) behind the hCMV-IE promoter of pLV.hCMV-IE.IRES.PurR.hHBVPRE (Neshati et al., 2014). To this end, the insert was combined with the 8122-bp SmaI×EcoRI fragment of pLV.hCMV-IE.IRES.PurR.hHBVPRE (Neshati et al., 2014). Recombinant plasmid construction was done with enzymes from New England Biolabs (Bioké, Leiden, the Netherlands) or Fermentas (ThermoFisher Scientific) using standard procedures or following the instructions provided with specific reagents. The plasmids were amplified in *Escherichia coli* GeneHogs (ThermoFisher Scientific) cells and purified using LabNed Plasmid Maxiprep Kits (ITK diagnostics, Uithoorn, the Netherlands).

LV production
LV particles were produced essentially as described previously (Liu et al., 2018) except that PEI MAX 40K (Polysciences Europe, Hirschberg an der Bergstraße, Germany) instead of PEI 25K was used as transfection agent and the polyethyleneimine-DNA complexes were left on the cells for only 4 hours.

LV transduction of hiPSC-VSMCs
The LV shuttle plasmid pLV.hCMV.-IE.GCaMP6f(+).IRES.PurR.hHBVPRE was used to express GCaMP6f in LUMC0054iCTRL hiPSC-NCCs P3. The LV shuttle plasmid pLV.hCMV.-IE.eGFP.IRES.PurR.hHBVPRE) was used to express pEGFP in LUMC0054iCTRL hiPSC-NCCs P3. Briefly, one day after seeding 40.000 cells/12-well on Matrigel-coated plates, hiPSC-NCCs were transduced with 2.5 µl viral particles in B(P)EL medium overnight. 96h post-transduction with complete NC differentiation medium infected cells were selected with 1 µg/mL puromycin (Sigma, P7255). After 4 days, remaining cells were expanded (1:3 ratio) Matrigel-coated plates then dissociated with 1xTrypLE Select and cryopreserved at passage number 3 (P3) using serum-free cryopreservation medium (CryoStor™CS10) (StemCell Technologies, 07930). Next, hiPSC-NC cells (hiPSC-NCCs) were differentiated into hiPSC-VSMCs as described previously with minor modifications (Halaidych et al., 2019a).

Flow cytometry analysis
hiPSC-NCCs were dissociated with 1xTrypLE Select and washed once with FACs buffer containing 10% FBS, and once with FACs buffer. CD271-BV421 (BD Biosciences, 562562, 1:100) surface antibody was used for the FACS staining. Analysis of samples was performed on the MACSQuant VYB (Miltenyi Biotec, 130-096-116).

Assessment of intracellular Ca^{2+} release in hiPSC-VSMCs
Intracellular Ca^{2+} release in hiPSC-VSMCs engineered to express GCaMP6f was performed as described previously (Halaidych et al., 2019a). Briefly, cells were seeded into a microfluidic chip (Vena8 Endothelial+, Cellix Ltd) coated with 50 µg/mL bovine fibronectin (Sigma) at density 10^4 cells/µL. Cells were kept in a CO_2 incubator for 3 hours before functional analysis. Single channel perfusion was enabled by a PC-controlled syringe pump (Mirus Evo Nanopump, Cellix). The microfluidic chip was placed into a live imaging chamber (+37°C, 5% CO2, humidified) mounted on a Leica AF6000

microscope. Image sequences of fluorescence were captured at 2 frames per second using a 10x magnification objective with 2x2 binning (spatial resolution: 2.28 µm/pix). First, basal fluorescence activity upon B(P)EL medium flow was captured. Then image capturing was paused and the inlet reservoir was filled with 1 µM Endothelin-I (Sigma) diluted in B(P)EL medium. Flow was applied again and simultaneous image capturing was continued.

Assessment of intracellular Ca^{2+} release in the VoC

Intracellular Ca^{2+} release upon medium refreshment and upon stimulation with the vasoconstrictor (ET-I) was analyzed on day 7 of VoC culture. Sequences of images prior to- (basal state) and after medium refreshment were captured using EVOS M7000 with a 10x objective. For the medium refreshment, medium from all ports of the microfluidic channel was first removed and gravity-driven flow was induced by the addition of 100 µl medium to the right media ports and 50 µl medium to left connecting media ports. After 30s, fluorescence activity of the whole microfluidic channel was captured. For real-time intracellular Ca^{2+} release upon stimulation with the vasoconstrictor, the microfluidic chip was placed into a humidified live cell imaging chamber (+37°C, 5% CO_2) and mounted on a DragonFly spinning disk microscope (Andor) with a 20x magnification objective on day 7 of culture. First, medium from all ports was removed and refreshed with 30 µl of EGM-2. After 30 min, basal fluorescence activity was captured for 5 seconds. Next, gravity-driven flow was induced by the addition of 60 µl EGM-2 or EGM-2 supplemented with 1.5 µM ET-I (Sigma) with a final concentration of 1 µM after the addition to the right medium ports containing 30 µl of EGM-2. Then, simultaneous image capturing was continued for 160 seconds. Image sequences of fluorescence were captured at 4 frames per second. After simultaneous image capturing, confocal images were acquired to create a 3D stack and processed using Imaris 9.5 software (Bitplane, Oxford Instruments).

Analysis of intracellular Ca^{2+} Release

Images sequences were processed using a freely available plugin ‘‘LC Pro’’ for ImageJ (https://imagej.nih.gov/ij/plugins/lc-pro/index.html) (Yip and Sham, 2012). Free open-source CellProfiler software (https://cellprofiler.org/) (Carpenter et al., 2006) was used to determine the total number of cells in a field of view. Output data were analysed as previously described (Halaidych et al., 2019a; Halaidych et al., 2019b).

Supplemental References

Carpenter, A.E., Jones, T.R., Lamprecht, M.R., Clarke, C., Kang, I.H., Friman, O., Guertin, D.A., Chang, J.H., Lindquist, R.A., Moffat, J.*, et al.* (2006). CellProfiler: image analysis software for identifying and quantifying cell phenotypes. Genome Biol *7*, R100.

Chen, T.W., Wardill, T.J., Sun, Y., Pulver, S.R., Renninger, S.L., Baohan, A., Schreiter, E.R., Kerr, R.A., Orger, M.B., Jayaraman, V.*, et al.* (2013). Ultrasensitive fluorescent proteins for imaging neuronal activity. Nature *499*, 295-300.

Halaidych, O.V., Cochrane, A., van den Hil, F.E., Mummery, C.L., and Orlova, V.V. (2019a). Quantitative Analysis of Intracellular Ca(2+) Release and Contraction in hiPSC-Derived Vascular Smooth Muscle Cells. Stem Cell Reports *12*, 647-656.

Halaidych, O.V., Freund, C., van den Hil, F., Salvatori, D.C.F., Riminucci, M., Mummery, C.L., and Orlova, V.V. (2018). Inflammatory Responses and Barrier Function of Endothelial Cells Derived from Human Induced Pluripotent Stem Cells. Stem Cell Reports *10*, 1642-1656.

Halaidych, O.V., Mummery, C.L., and Orlova, V.V. (2019b). Quantifying Ca(2+) signaling and contraction in vascular pericytes and smooth muscle cells. Biochem Biophys Res Commun *513*, 112-118.

Hotaling, N.A., Bharti, K., Kriel, H., and Simon, C.G., Jr. (2015). DiameterJ: A validated open source nanofiber diameter measurement tool. Biomaterials *61*, 327-338.

Liu, J., Volkers, L., Jangsangthong, W., Bart, C.I., Engels, M.C., Zhou, G., Schalij, M.J., Ypey, D.L., Pijnappels, D.A., and de Vries, A.A.F. (2018). Generation and primary characterization of iAM-1, a versatile new line of conditionally immortalized atrial myocytes with preserved cardiomyogenic differentiation capacity. Cardiovasc Res *114*, 1848-1859.

Neshati, Z., Liu, J., Zhou, G., Schalij, M.J., and de Vries, A.A. (2014). Development of a lentivirus vector-based assay for non-destructive monitoring of cell fusion activity. PLoS One *9*, e102433.

Orlova, V.V., Drabsch, Y., Freund, C., Petrus-Reurer, S., van den Hil, F.E., Muenthaisong, S., Dijke, P.T., and Mummery, C.L. (2014a). Functionality of endothelial cells and pericytes from human pluripotent stem cells demonstrated in cultured vascular plexus and zebrafish xenografts. Arterioscler Thromb Vasc Biol *34*, 177-186.

Orlova, V.V., van den Hil, F.E., Petrus-Reurer, S., Drabsch, Y., Ten Dijke, P., and Mummery, C.L. (2014b). Generation, expansion and functional analysis of endothelial cells and pericytes derived from human pluripotent stem cells. Nat Protoc *9*, 1514-1531.

Rostovskaya, M., Fu, J., Obst, M., Baer, I., Weidlich, S., Wang, H., Smith, A.J., Anastassiadis, K., and Stewart, A.F. (2012). Transposon-mediated BAC transgenesis in human ES cells. Nucleic Acids Res *40*, e150.

Yip, K.P., and Sham, J.S. (2012). Tracking stars: automated two-dimensional analysis of Ca(2)(+) events. Focus on "Automated region of interest analysis of dynamic Ca(2)(+) signals in image sequences". Am J Physiol Cell Physiol *303*, C233-235.

Zhang, M., D'Aniello, C., Verkerk, A.O., Wrobel, E., Frank, S., Ward-van Oostwaard, D., Piccini, I., Freund, C., Rao, J., Seebohm, G.*, et al.* (2014). Recessive cardiac phenotypes in induced pluripotent stem cell models of Jervell and Lange-Nielsen syndrome: disease mechanisms and pharmacological rescue. Proc Natl Acad Sci U S A *111*, E5383-5392.
